# EEG signatures of cognitive and social development of preschool children–a systematic review

**DOI:** 10.1371/journal.pone.0247223

**Published:** 2021-02-19

**Authors:** Supriya Bhavnani, Georgia Lockwood Estrin, Rianne Haartsen, Sarah K. G. Jensen, Teodora Gliga, Vikram Patel, Mark H. Johnson

**Affiliations:** 1 Sangath, New Delhi, India; 2 Centre for Brain and Cognitive Development, Birkbeck, University of London, London, United Kingdom; 3 Boston Children’s Hospital, Harvard Medical School, Boston, MA, United States of America; 4 School of Psychology, University of East Anglia, Norwich, United Kingdom; 5 Harvard T.H. Chan School of Public Health, Boston, MA, United States of America; 6 Department of Psychology, University of Cambridge, Cambridge, United Kingdom; Australian Research Council Centre of Excellence in Cognition and its Disorders, AUSTRALIA

## Abstract

**Background:**

Early identification of preschool children who are at risk of faltering in their development is essential to ensuring that all children attain their full potential. Electroencephalography (EEG) has been used to measure neural correlates of cognitive and social development in children for decades. Effective portable and low-cost EEG devices increase the potential of its use to assess neurodevelopment in children at scale and particularly in low-resource settings. We conducted a systematic review aimed to synthesise EEG measures of cognitive and social development in 2-5-year old children. Our secondary aim was to identify how these measures differ across a) the course of development within this age range, b) gender and c) socioeconomic status (SES).

**Methods and findings:**

A systematic literature search identified 51 studies for inclusion in this review. Data relevant to the primary and secondary aims was extracted from these studies and an assessment for risk of bias was done, which highlighted the need for harmonisation of EEG data collection and analysis methods across research groups and more detailed reporting of participant characteristics. Studies reported on the domains of executive function (n = 22 papers), selective auditory attention (n = 9), learning and memory (n = 5), processing of faces (n = 7) and emotional stimuli (n = 8). For papers investigating executive function and selective auditory attention, the most commonly reported measures were alpha power and the amplitude and latency of positive (P1, P2, P3) and negative (N1, N2) deflections of event related potential (ERPs) components. The N170 and P1 ERP components were the most commonly reported neural responses to face and emotional faces stimuli. A mid-latency negative component and positive slow wave were used to index learning and memory, and late positive potential in response to emotional non-face stimuli. While almost half the studies described changes in EEG measures across age, only eight studies disaggregated results based on gender, and six included children from low income households to assess the impact of SES on neurodevelopment. No studies were conducted in low- and middle-income countries.

**Conclusion:**

This review has identified power across the EEG spectrum and ERP components to be the measures most commonly reported in studies in which preschool children engage in tasks indexing cognitive and social development. It has also highlighted the need for additional research into their changes across age and based on gender and SES.

## Introduction

The importance of making a concerted global effort towards optimising early child development is rapidly being recognised, particularly as child survival increases due to the successful reduction in infant and child mortality rates across the world. Using stunting and poverty as indicators, Lu and colleagues demonstrated that over 200 million children in low and middle income countries (LMICs) are at risk of suboptimal development [1]. Another study using care-giver report data from 35 LMICs suggests that one in every three preschool-age children are failing to meet expected cognitive or social developmental milestones [2]. Cognitive abilities include learning and memory, selective visual and auditory discrimination and executive function; social abilities can be indexed by how children process facial and emotional stimuli. These domains of development lay the foundation for learning and therefore readiness for school, with delayed or suboptimal development of these abilities negatively impacting academic performance [3,4].

Key to ensuring that all children thrive is the early identification of those not following a typical developmental trajectory, and their subsequent timely referral to interventions. The most widely used approach to assess neurodevelopment is behavioural observations by specialists. Given the scarcity of clinical professionals in LMICs, it is essential to create and validate efficient methods that are objective, amenable for administration by trained non-specialist workers and therefore scalable in multiple low resource settings [5,6]. Neurophysiological methods like electroencephalography (EEG) offer complementary methods to assess brain development in children as it is a non-invasive, direct measure of brain activity with high temporal resolution. In addition to laboratory grade equipment used in most EEG studies, low-cost, portable EEG devices have recently become available on the market. Some companies also offer cloud-based analysis of the data, removing the need for expertise on site. These advances present an opportunity to examine the potential use of EEG at scale in the future [7].

Given that brain plasticity (ability to adapt to environmental circumstances) is at its peak in early childhood, interventions to optimise child development implemented during preschool years are known to be most effective in improving developmental outcomes [8–10]. Intervening in the early years also provides the highest return on investments, further strengthening the argument in favour of early identification of children at risk for not attaining their full developmental potential [11].

A significant amount of research has been done to establish trajectories of cognitive and social development. However, due to disparities in research funding, these studies, which require considerable sample sizes and longitudinal follow up, have largely been restricted to children from high-income countries (HICs). This is despite the fact that a disproportionately greater number of children at risk of not attaining their full developmental potential reside in LMICs. Given emerging evidence that signatures of brain development differ across cultures, there is an urgent need to capture a broader range of developmental trajectories globally including underserved populations [12]. This is an essential first step to identification of children who are developing sub-optimally and improvement of their individual prospects, resulting, in the long term, in lifting people out of poverty to break the vicious cycle of intergenerational transmission of disadvantage [13].

To this end, it is valuable to synthesise the existing knowledge that EEG studies, which have been used to assess neural correlates of cognitive and social developmental processes such as visual attention and memory for decades albeit in HICs, has generated [14–17]. A range of measures have been developed to examine: a) the timing (latency) and amplitude of event-related potentials (ERPs), time-locked brain activity in response to a stimulus [18]; or b) continuous brain activity, either during a task or at rest (called resting state), examining the synchronisation of oscillations via spectral power and connectivity [19]. Accumulating evidence highlights the potential of EEG recorded during resting state to identify children faltering in their development [16,20] or those with neurodevelopmental disorders such as autism spectrum disorders (ASD), attention deficit hyperactivity disorder (ADHD) or learning-disability [21–27]. Recent systematic reviews have also focused on establishing the prognostic accuracy of resting-state EEG recorded in preterm infants in predicting neurodevelopmental outcomes [28,29]. Some efforts have also been made to synthesise the vast body of EEG literature to isolate resting-state EEG measures that can serve as signatures of cognitive and social development in preschool children [30].

However, there are limited reviews consolidating measures that are derived from EEG recordings done while preschool children, aged 2–5 years, are engaged in tasks designed to measure cognitive and social development [31], perhaps due to the challenges in assessing children of this age. Therefore, in an effort to identify neural correlates that may reflect the developmental status of key cognitive and social abilities in preschool children, we conducted a systematic review of the EEG literature to synthesise existing knowledge across studies. Our primary aim was to identify task-related EEG measures that indexed cognitive and social development in children aged 2–5 years.

Furthermore, EEG measures have been demonstrated to change over the course of development [14,32], with evidence of non-linear brain development emerging from early seminal EEG studies in the 1980s-90s conducted by Thatcher and colleagues [33–35]. Interestingly, the evidence of differences based on gender is mixed [36,37]. Some studies also indicate differences in EEG measures based on socioeconomic status of children [38,39]. Gaining clarity on EEG differences based on gender and SES are particularly relevant in the context of the children from LMICs who are more likely to grow up in poverty, with girls often receiving a disproportionately low share of scant resources [40]. Our secondary aim was thus to identify how such EEG measures differ across a) the course of development within this age range, b) gender and c) socioeconomic status.

## Methods

### Protocol

**Fig 1:**
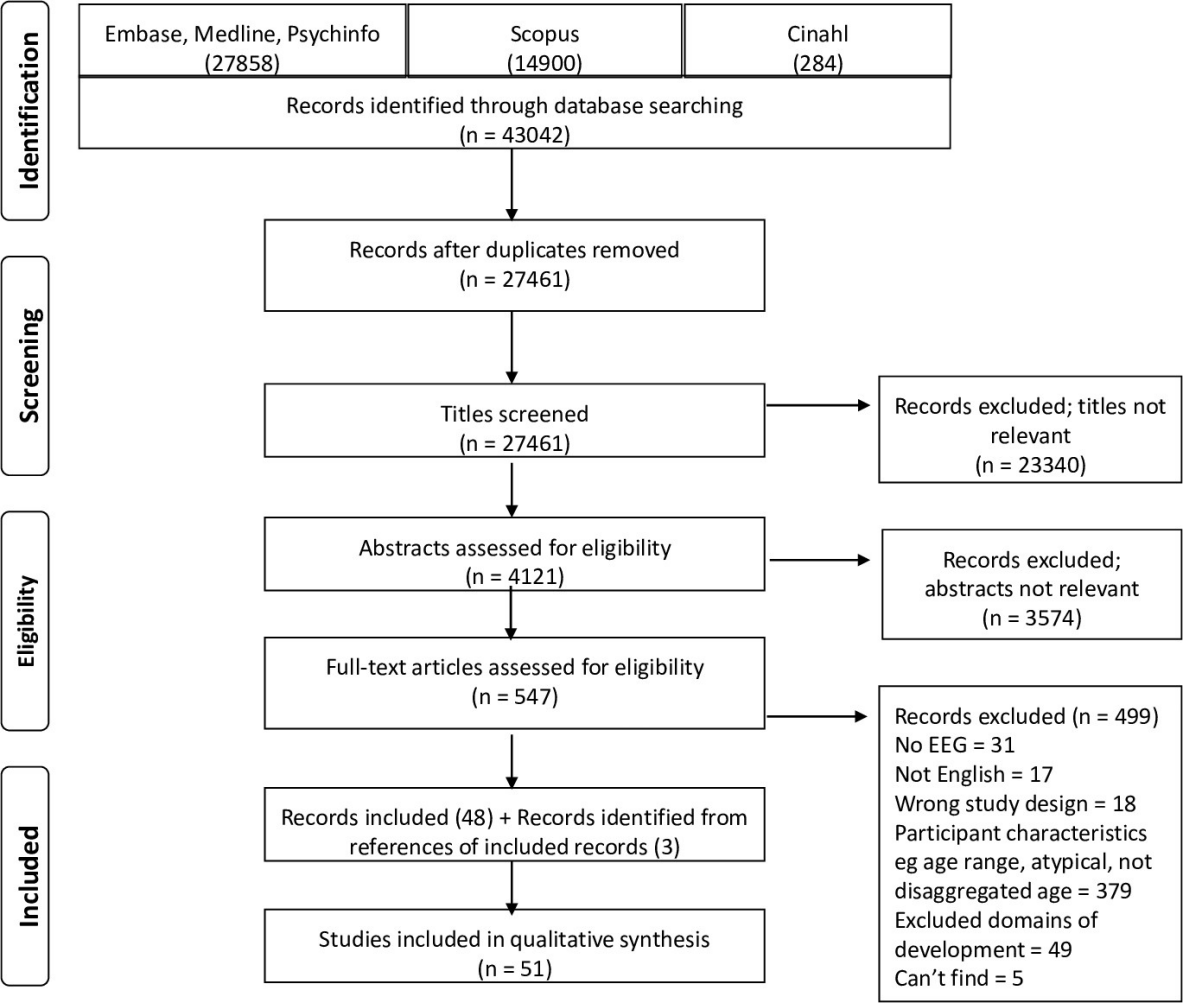
PRISMA flowchart—summary of the results of the search conducted in this review.

This systematic review was conducted following PRISMA guidelines (http://www.prisma-statement.org) and the results are summarised in a PRISMA flowchart in Fig 1. A comprehensive literature search was conducted on five databases—Embase, Medline and Psycinfo (searched on 12/01/2018, updated on 15/05/2020) yielded 27858 records, Scopus (searched on 16/02/2018, updated on 15/05/2020) yielded 14900 records and Cinahl (searched on 23/02/2018, updated on 15/05/2020) yielded 284 records. The search terms included EEG, cognition, attention, social development and child development and are detailed in S1 Table. These 43042 records contained 15581 duplicates which were removed. Reviewers (SB and GLE) split the remaining 27461 records equally. All 27461 records were screened first at the title level and any undecided papers were discussed between reviewers. Abstract screening was completed on 4121 records, and inter-rater reliability was established between reviewers SB and GLE at this stage. Shared reference manager tool (Endnote and Rayyan) libraries were used to consult each other about inclusion or exclusion of papers throughout the process of review. Each reviewer classified approximately 10% of the other’s papers while being blinded to the other’s decisions. Cohen’s kappa was determined to be 0.63 (95% CI: 0.52, 0.74) representing ‘good’ reliability. All conflicting decisions were discussed and resolved, and any unresolved papers were discussed with co-author RH until consensus was reached. Based on the screening of abstracts, 547 records made it to the full text review and, based on the criteria below, 48 were categorised into ‘included’ while 499 were ‘excluded’. In order to ensure comprehensiveness of the database search, bibliographies of recent included studies were reviewed for relevance to our research question. Three relevant records were added through this process resulting in final inclusion of 51 papers.

### Inclusion/Exclusion criteria

Articles were excluded if they 1) were not published within 3 decades prior to the search date, 2) were not published in English language or peer-reviewed journals, 3) were case or series of case studies (number of participants ≤10), 4) reported results of interventions, 5) did not contain participants within the target age range of 2–5 years i.e. 24–72 months, 6) included participants beyond the target age range and did not disaggregate results by age, 7) included participants with atypical development including any diagnosed mental and neurodevelopmental disorders or cognitive delays, physical disabilities or genetic disorders, 8) included participants at risk of atypical development due to known risk factors such as preterm birth, pre- and perinatal infections and maternal conditions like diabetes or depression, 9) were conducted while participants were in resting state, asleep/unconscious, anxious, in fear, pain, or experiencing a headache and 10) assessed lower level sensory processing such as vision and hearing, other domains of development such as language, and motor, or academic skills like mathematic/arithmetic, reading/comprehension. Studies reporting assessment of cognitive and social domains of executive function, selective auditory attention, learning and memory, processing of faces and emotional stimuli as defined by the papers, were included.

### Data extraction

A data extraction table was created for the following features of included papers: a) *publication* details such as income level of the country in which the study was conducted and sample size; b) *participant* characteristics such as age, demographic information including parental education and income, recruitment strategy, inclusion/exclusion criteria and reasons for loss of participants; c) *EEG device* characteristics such as brand, number of electrodes and sampling rate; d) *study procedure* details like the type of cognitive domain assessed, task used and setup information; e) *data pre-processing* steps of filtering, artefact identification and rejection, segmentation of EEG signal, regions and time windows of interest; f) *data analysis* methods, including statistical methods, significant results and conclusions relevant to the primary and secondary aims of this review, and finally g) *limitations* of the study acknowledged by the authors. Reviewers SB and GLE split the included records equally and extracted data independently. The data that has been extracted was then synthesised by them together in close consultation with co-author RH.

### Assessment of risk of bias

The KMet quality appraisal checklist [41], which was created in response to the need for standardised quality assessment criteria applicable for evaluating primary research studies from a variety of fields, was piloted and 11 of the 14 questions were adapted in consultation with co-author SJ for use in this study, along with two additional questions (see S2 Table for list of 13 quality appraisal questions). Reviewers SB and GLE appraised all studies together by achieving consensus through discussions. Studies could score either 2 for ‘yes’ (high quality), 1 for ‘partial’ or 0 for ‘no’ (low quality) for each quality appraisal question. For the question on appropriate sample size (question 8), studies were scored ‘yes’ if they included 20 or more participants in each analysis group (e.g. age groups), ‘partial’ if total participant number was 20 or more but analysis group number was less than 20 and ‘no’ if data from less than 20 participants was analysed. Percentage of yes, partial and no were calculated to provide a graphical summary of the appraisal of all included studies.

## Results

### Characteristics of included studies

#### Study participants

The 51 studies included in this review represent a total of 2123 participants within the target age range of 2–5 years. Most of the participants of included studies were aged between 4–5 years (S1 Fig and Table 1) with fewer studies (9/44) conducted with younger (2-3-year old) children. The participants of these studies covered an age range of over 1.5 years. Almost half (24/51) of the included studies report developmental changes in EEG measures by comparing cross-sectional data from preschool children with other ages ranging from infants to adults. All studies specified the distribution of gender amongst their participants, however only eight report on the impact of gender on their results.

**Table 1:** Details of included papers: Sample size, mean age, task name, EEG metric and results in relation to the cognitive ability, age, gender and socioeconomic status.

Study ref	Sample size Age in years, mean (SD) / range	Task name	EEG measure	Results
Executive function: visual attention, working memory and inhibitory control				
Lahat et al. (2009) [42]	N = 37 European-Canadian; Mean: 5.38 (0.41) (n = 20) Chinese-Canadian; Mean: 5.18 (0.65) (n = 17)	Go/No-Go Task	Amplitude and latency of N2 ERP component	An asymmetrical pattern of scalp lateralization (to the right for No-go trials and to the left for Go trials) suggested that a cortical generator in left and right VLPFC may contribute more to Go and No-Go N2s respectively. Other changes: 1. Larger N2 amplitude in Chinese-Canadian than European-Canadian children 2. The asymmetric pattern of lateralization was more pronounced for Chinese-Canadian children than for European-Canadian children.
Chevalier et al. (2014) [43]	N = 30 Mean: 5.7 (0.5)	Go/No-Go Task	Peak amplitude and latency of Lateral Frontal Negativity (LFN) of ERP component	1. ERP data showed a left-lateral frontal negativity (LFN) 2. LFN amplitude greater for partial and successful No-Go than Go responses. 3. Longer LFN latencies for partial relative to successful No-Go and successful Go responses 4. Children with longer LFN latencies on successful Go trials had slower response speed
Rahman et al. (2017) [44]	N = 31 Mean: 5.67 (0.25)	Go/No-Go task	Mean amplitude and peak latency of P1, N2 and P3 ERP components	1. Time pressure modulated the P1, with P1 amplitude being greater for children in the slow condition than the fast condition. No difference between fast and slow conditions were found for ERP markers of response inhibition. 2. Relative to Go trials, No-go trials elicited longer N2 latencies at left frontal electrodes and enhanced N2 and P3 at midline electrode sites.
Hoyniak et al. (2018) [45]	N = 107 30 months Mean: (n = 52) 36 months Mean: (n = 50) 42 months Mean: (n = 59)	Go/No-Go task	1. N2 2. N2 effect = NoGo N2 amplitude minus Go N2 amplitude	1. The N2 elicited to NoGo trials was more negative than to Go trials 2. Better performance on NoGo trials was associated with a smaller difference between Go and NoGo N2 amplitudes 3. Higher levels of parent reported inhibitory and attentional control was associated with smaller NoGo N2 amplitudes
Brooker (2018) [46]	N = 119 3.5-year olds Mean: 3.59 (0.15) (n = 108) 4-year olds Mean: 4.56 (0.15) (n = 97)	Go/No-Go task	Error-related negativity (ERN)	Change across age: No change in ERN from age 3 to age 4 Change across SES: 1. In high SES and high maternal sensitivity, ERN at age 3 predicted ERN at age 4 2. In low SES, ERN at age 3 does not predict ERN at age 4 No significant results in relation to paternal sensitivity
St John (2019) [47]	N = 69 Mean: 5.04 (0.27)	Go/No-Go task	P3b amplitudes	1. P3b amplitudes were larger on no-go compared to go trials Change across gender: No significant effect of gender, but pattern suggested females had larger P3b amplitudes on go trial Change across SES: A higher household income level was associated with larger P3b amplitudes
Rueda et al. (2004) [48]	N = 32 Mean: 4.33 (0.18); (n = 14) (Also 23-year old adults)	Flanker task	1. Peak latency and peak amplitude of N1, N2 and P3 ERP components 2. Amplitude of late positive component (LPC)	1. Overall reaction time correlated negatively with P3 amplitude 2. Larger P3 amplitude and longer latency in parietal regions for incongruent than congruent trials 3. Less positive amplitude of the LPC during incongruent than congruent trials in frontal (mostly pre-frontal) sites. Change across age: 1. Larger N1 and N2 amplitudes for children than adults, P3 amplitude equivalent in the two groups. 2. Longer N1, N2, P3 latency in children compared to adults. The difference was greater in the later components 3. Effect on the P3 amplitude and latency is lateralized to the left in adults and right in children.
Begnoche et al. (2016) [49]	N = 41 Mean: 4.59 (0.13) BIS BAS association (n = 26) Predictive EEG (n = 21)	Flanker task	1. Error related negativity (ERN) 2. ERN difference wave (ΔERN) 3. Alpha (6-10Hz) asymmetry	1. Larger ERN amplitude to incorrect than correct trials in frontal and central sites 2. Demonstrated a link between a neural index of BIS (i.e., the ERN) and BAS (i.e., hemispheric asymmetry) at 4.5 years age at parietal electrode sites
Ruberry et al. (2016) [50]	N = 80 Range: 4.5–4.83 (n = 62) 5.25–5.58 (n = 56) Flanker task (n = 61) Frogs/fish task (n = 72) Both tasks (n = 53)	1. Frogs/fish task 2. Flanker task	Peak amplitude of N2 and P3 ERP components	1. N2 difference score for Go versus No-Go trials was positively correlated with performance on the frogs/fish task, and the broader executive control battery. 2. P3 amplitude during congruent and incongruent trials was negatively correlated to performance on the flanker task and the broader executive control battery Change across SES: Income, cumulative risk (risk factors) and financial security were not related to amplitude of the N2 or P3 on either EEG task
Morasch & Bell (2011) [51]	Mean: 2.08 (0.05); Electrode compliance (n = 81) A-not-B and Crayon delay (n = 58)	1. A-not-B task with invisible displacement 2. Crayon delay task 3. Electrode acceptance	Alpha (6 to 9 Hz) power	Task-related inhibitory control performance on the conflict task as well as baseline-to-task decreases in lateral frontal EEG power accounted for 29% variance in inhibitory control. Change across gender: No effect of gender
Espinet et al. (2012) [52]	N = 99 Mean: 3.48 (0.36)	Dimensional Change Card Sort Task (DCCS)	Peak amplitude and peak latency of N2 ERP component	1. Compared to children who perseverated on the DCCS, children who switched flexibly had smaller N2 amplitudes, but no difference in N2 latency, during the post-switch phase and pre-switch trials of the task 2. N2 response originated in cingulate and orbitofrontal regions.
Blankenship et al. (2018) [53]	N = 40 Mean: 4.46 (0.30)	Dimensional Change Card Sort Task (DCCS) and Semantic Future Thinking Task (SFT)	Frontal EEG power: alpha (6–9 HZ) frequency band	1. EF performance was not correlated with frontal EEG during the SFT task 2. Medium to high frontal EEG power values moderates the relation between executive functioning and semantic future thinking performance, but not low level frontal EEG power values
Lo et al. (2013) [54]	N = 43 Mean: 5.49 (0.23); (n = 22, 10 for ERP) (Also 21 6-year olds)	Stop signal task	1. Amplitude of N2 ERP component 2. Power for defined bands between 2–65 Hz	1. Larger N2 amplitudes in unsuccessful than successful trials 2. Increased alpha and right frontal beta power in successful trials. Change across age: 1. N2 effect (larger N2 amplitude in unsuccessful than successful trials) unable to account for behavioral improvement between 5 and 6 year olds. 2. Power change in beta and lower gamma band increased with age from 5 to 6 years
Elke & Wiebe (2017) [55]	N = 39 Mean: 5.33; Range: 4.67–5.92 (n = 17) (Also 7–8 year olds)	Ocean Sort Task	1. Peak latency and amplitude P2 and P3 ERP components 2. Amplitude of slow wave	1. Switch trials were associated with larger cue-P3 amplitudes than stay trials at all analyzed electrode clusters and had larger slow wave amplitudes at central, parietocentral, parietal and right parietocentral electrode clusters. 2. Stimulus-P2 latencies were shorter in switch trials than in stay trials at the midline central and midline parietocentral electrode clusters Change across age: Cue P2 amplitudes were larger for 7–8 than 4-5-year old children, regardless of switch condition in right and midline parietocentral electrodes. No other ERP components differed.
Wolfe & Bell (2004) [56]	N = 20 Range: 4.33–4.67	1. Stroop-like day–night task 2. Yes–no task	Alpha (6 to 9 Hz) power	Three predictors of WMIC group: The PPVT-III language measure, the left medial frontal EEG power (F3), and the approach/anticipation dimension of temperament. Together, these three variables were able to correctly classify 90% of the children to high and low WMIC groups
Bell & Wolfe (2007) [57]	N = 18 Range: 4.33–4.67 (Also 0.67–0.73)	1. Stroop-like day–night task 2. Yes–no task	1. Alpha (6 to 9 Hz) power 2. Intrahemispheric coherence	Change across age: 1. At 8 months of age, working memory is associated with increases in EEG power from baseline to task across the entire scalp but at medial frontal only at 4.5 years. 2. Decreases in EEG coherence from baseline to task across all electrode pairs at 8 months but only the medial frontal/posterior temporal and medial frontal/occipital electrode pairs exhibited increases in EEG coherence at 4.5 years
Wolfe & Bell (2007) [58]	N = 39 Range: 3.42–3.58 (n = 9) 3.92–4.08 (n = 13) 4.42–4.58 (n = 17)	1. Stroop-like day–night task 2. Yes–no task	Alpha (6 to 10 Hz) power	Left and right medial frontal regions are valuable for explaining variance in WMIC performance (composite score created across tasks) in 4 year olds and marginally for the 3.5 -year-old. Change across age: 1. 6–10 Hz power values at age 3.5 is higher than 4 and 4.5 year olds 2. Baseline-to-task 6–10 Hz power increases at four regions (frontal pole, medial frontal, anterior temporal, and posterior temporal) at age 4 but three regions (frontal pole, medial frontal—left, and posterior temporal) at age 4.5 years
Wolfe & Bell (2007) [59]	N = 18 Range: 4.33–4.67 (Also 0.67–0.73)	1. Stroop-like day–night task 2. Yes–no task	Alpha (6 to 9 Hz) power	Increased frontal brain electrical activity, low temperament scores (i.e. low approach/anticipation behaviours), and increased language scores predicted good performance on working memory at age 4.5-years. Change across age: Negative correlation between infant and child frontal EEG power
Watson & Bell (2013) [60]	N = 64 Mean: 3.11 (.08)	1. Less is more 2. Hand game 3. Stroop-like day-night task	Alpha (6 to 9 Hz) power	Medial frontal baseline-to-task changes in EEG activity (25%), along with language, temperament-based IC, and maternal education accounted for 39% of the variance in Hand Game performance.
Wolfe & Bell (2014) [61]	N = 122 recruited, 101 analysed Mean: 4.07 (.26) Shy (n = 59) non-shy (n = 63)*	1. Stroop-like day–night task 2. Yes–no task	Alpha (6 to 9 Hz) power	Increase in medial frontal EEG power from baseline-to-task for high EF performers (shy and non-shy). Shy/low EF performers also demonstrated this increase, but the non-shy/low EF group did not. For the medial parietal region, only the shy children (high and low EF performers) showed an increase in power from baseline-to-task; and for the shy/high EF group, left hemisphere power was greater than the right during baseline and task.
Cuevas et al. (2016) [62]	N = 144 Mean: 4.07 (0.06)	1. Stroop-like day-night task 2. Non-Stroop-like day-night task	Alpha (6 to 9 Hz) power	4-year-olds exhibit increases in 6–9 Hz EEG power in response to added executive processing demands (i.e., “Stroop-like” vs. “non-Stroop” day-night tasks) Change across gender: 1. Although both sexes exhibited significant changes for lateral and medial frontal, temporal, and lateral parietal regions, boys also exhibited changes for medial parietal and occipital regions. 2. Girls also exhibited higher overall levels of 6–9 Hz EEG power than boys.
Swingler et al. (2011) [63]	N = 96 Mean: 4.2 (.3); Power (n = 77) Coherence (n = 76)	1. Working Memory Span 2. Pick the Picture 3. Spatial Conflict Arrows 4. Something’s the Same 5. Silly Sounds Stroop 6. Animal Go/No-Go	1. Alpha (6 to 9 Hz) power 2. Intrahemispheric coherence	Decrease from baseline to task engagement in EEG coherence, but not EEG power, were significantly related to performance on the EF battery
Selective auditory attention				
Bartgis et al. (2003) [64]	N = 36 Mean: 5.7; Range: 5–5.92 (n = 12) (Also 7 and 9 year olds)	Selective auditory attention (adapted from Hansen and Hillyard, 1980)	Mean amplitude of P3 and Nd ERP components	Change across age: 5- year-olds have an inability to attend to channels differentially (demonstrated by the lack of an Nd wave) and an inability to select the relevant stimulus within the appropriate channel (demonstrated by equal amplitude of the P3 for both attended and ignored targets) which increase by 7 years age
Sanders et al. (2006) [65]	N = 39 Mean: 4.75; Range: 3.33–5.92	Selective auditory attention (adapted from Coch et al., 2005)	Mean amplitude of 100–200 ms, 200–300 ms, and 300–450 ms epochs	1. Children showed a broad positivity rather than the positive-negative-positive ERP oscillation in response to auditory onsets 2. The attention effect (attended—unattended) extended from 100–300 ms and was positive in polarity
Pesonen et al. (2010) [66]	N = 15 Mean: 2.8 (0.3)	Selective auditory attention (adapted from Pakarinen et al, 2004)	Amplitude of P3a ERP component	1. Higher level of temperamental effortful control was associated with larger P3a responses to repeated novel, attention-catching sound. 2. Higher negative emotionality was related to smaller P3a responses to repeated novel sounds. 3. More synchronous parent–child interaction was associated with larger P3a responses to repeated novel sounds. Change across age: Higher extraversion was associated with larger P3a responses to non-repeated animal and mechanical sounds, and only in the age-adjusted model.
Sanders and Zobel (2012) [67]	N = 18 Mean: 5.0 (0.6) (Also 21–31 year old adults)	Selective auditory attention	Mean amplitudes of P1-N1 complex	Probes elicited a broadly distributed positivity that peaked around 100 ms after onset rather than the more mature positive-negative-positive oscillation. However, there was some indication of a first negative peak in children by 150 ms. Change across age: Attended sounds elicit a larger negativity by 80 ms in children and adults indicating that mechanisms by which attention modulates perceptual processing are in place by 4–5 years age.
Strait et al. (2014) [68]	N = 24 Range: 3-5years; Response variability (n = 15) (Also 7–13-year-olds and 18–35-year-old adults)	Selective auditory attention (adapted from Coch et al., 2005)	1. Mean amplitudes of P1-N1 complex 2. Response variability over the first 300 ms post-stimulus onset	No impact of attention on cortical responses Change across age: Although preschoolers, school-aged children and adults have equivalent response variability to attended speech, only school-aged children and adults have a distinction between attend and ignore conditions. Preschoolers, on the other hand, demonstrate no impact of attention on cortical responses.
Karns et al. (2015) [69]	N = 88 Mean: 4.8 (0.6) (n = 20) (Also 10, 13, 16 and 18–26 year olds)	Selective auditory attention	Latency and mean amplitude of P1, early N1, P2 ERP components	For 3–5-year-olds the attention effect (attended minus unattended) elicited a broad positivity Change across age: In early childhood, auditory ERPs consist of a broad positivity from 100 to 300 ms, while the P1-N1 complex that is characteristic of adults does not emerge until early adolescence.
Isbell et al. (2016) [70]	N = 124 Mean: 4.5 (0.54)	Selective auditory attention	Mean amplitude difference between 100-200ms post stimulus onset	Larger, more positive attention effects (ERP responses to attended minus unattended condition) over the anterior and central electrode locations were associated with superior nonverbal IQ performance in children from low SES.
Wray et al. (2017) [71]	N = 47 High SES Mean: 4.23 (.12) (n = 14) Low SES Mean: 4.29 (.07) (n = 33) Low SES follow up Mean: 5.55 (.07) (n = 33)	Selective auditory attention as in Isbell, Hampton Wray, et al., 2016; Karns et al., 2015; Neville et al., 2013	Mean amplitude of P1, N1 and N2 ERP components	Change across SES: At age four, the higher but not lower SES group exhibited a significant attention effect (larger ERP responses to attended minus unattended condition). At age five, the lower SES group exhibited a significant attention effect comparable in overall magnitude to that observed in the 4-year-old higher SES group
Giuliano et al. (2018) [72]	N = 104 Mean: 4.31 (0.54)	Selective auditory attention as in Karns et al., 2015; Neville et al., 2013	Amplitude of positive ERP component (150–200 ms post stimulus onset)	Change across SES: Increased socioeconomic risk was associated with larger positive amplitudes elicited by distracting sounds
Learning and memory				
Marshall et al. (2002) [14]	N = 20 4 year olds (Also 17 22-year old adults)	Old (previously shown) vs new pictures of objects	Mean amplitudes for 300- to 600-msec, 600- to 900-msec, and 900- to 1,500-msec time windows. Different time windows for the adult sample	More positive-going ERPs (old-new effect) in response to pictures correctly classified as old compared with pictures correctly classified as new Change across age: 1. The old–new effect was displayed by 4-year-olds in the 900- to 1,500ms latency region while in adults it was observed as early as 450ms after stimulus onset 2. In 4-year olds, the old-new effect was stronger over the right versus the left hemisphere while in adults it was in both the right and left hemispheres.
Riggins et al. (2009) [73]	N = 48* Mean: 3 (0.05) (n = 22)* 4 (0.03) (n = 26)*	2 lab visits 1 week apart. Stimuli comprised digital photographs of a woman’s hand completing novel event sequences and those observed in behavioral testing	Amplitude and latency of negative middle latency component and Positive Slow Wave (PSW)	Item recall was associated with larger amplitude of a middle latency component, whereas recall for items in the correct temporal order was associated with larger PSW amplitude Change across age: Shorter latencies and smaller amplitudes/ decreased positive slow wave activity in 4 than the 3-year-old children.
Riggins & Rollins (2015) [74]	N = 59 Mean: 3.28 (.13) (n = 18) 4.28 (.15) (n = 18) 5.28 (.13) (n = 23)	2 lab visits 1–2 days apart. Stimuli comprised old items with and without contextual details and new items.	Amplitude of Negative component (Nc) and Positive Slow Wave (PSW)	Change across age: Amplitude of Nc and PSW to new items was greater than amplitude to old items recalled without contextual details, with amplitude to old items recalled with contextual details in between. This effect to items recalled with contextual details increased between 3 and 4 years, and the effect to items recalled without contextual details was greatest in 5-year-old children.
Canada et al. (2019) [75]	N = 61 Mean: 6.17 (1.28) Younger children: less than median of 5.86 years	Matching previously shown items with their relevant characters	Amplitude of Negative component (Nc) and Late Slow Wave (LSW)	Change across age: 1. No association between Nc and performance on memory task or age 2. Greater difference in mean LSW amplitude between source conditions for younger children compared to older children and was specific to younger, low-performing participants. Specifically, at parietal leads, mean amplitude was more negative for the source correct versus source incorrect condition
Meyer et al. (2014) [76]	N = 12 Mean: 2.56 (0.01)	Picture matching based on feedback	Feedback locked ERP per outcome category of correct and incorrect first turns	1. More pronounced negativity following incorrect than correct outcomes in 2.5-year olds 2. Larger electrophysiological difference between correct and incorrect first turns was associated with better behavioral performance on second turns
Face processing				
Taylor et al. (2001) [77]	N = 128 Mean: 4.7 (0.3); (n = 15) (Also 6–7, 8–9, 10–11, 12–13 and 14–15 year olds, and 28 year old adults)	1. Eyes vs faces 2. Scrambled faces 3. Inverted vs upright faces	Amplitude and latency of N170 ERP components	1. N170 amplitude was larger to facial than non-face stimuli in 4-5-year olds 2. N170 amplitude to eyes was much larger and at shorter latencies than faces in 4–5 year-olds Change across age: 1. Eyes, upright faces and inverted faces evoked N170s across age groups, whereas scrambled faces and non-face stimuli evoked very small N170s 2. N170 had much longer latencies in 4–5 year old children than in adults 3. The peak of N170 shifted from the superior to inferior electrodes from childhood to adolescence 4. Larger N170s over the right hemisphere for eye stimuli across all age groups. N170 to faces and inverted faces was usually larger over the left than right hemisphere in 4–5 year olds as opposed to adolescents and adults 5. P1 latency decreased with age and was shorter for the faces across all age groups 6. P1 amplitude increased in childhood and gradually declined over the teenage years Change across gender: N170 had shorter latencies and larger amplitudes to all stimuli in females than males but this effect was mostly driven by the older age groups
Peykarjou et al. (2013) [78]	N = 35 Mean: 3.42 (0.08)	1. Infant vs adult 2. Upright vs inverted	Amplitude and latency of P1, N170 and P400 ERP components	1. Shorter P1 latency and enhanced P400 amplitude for inverted faces. 2. Larger P1 amplitude in response to adult than infants faces and largest in the midline compared to right and left regions 3. N170 amplitude was larger in response to adult than infants faces 4. Sibling age at test and P1 amplitude correlated negatively for adult and newborn faces
Lochy et al. (2019) [79]	N = 34 Mean: 5.51; Range: 5.01–5.94	Fast Periodic Visual Stimulation (Rossion, 2014)	Signal to noise response (SNR) spectra	The face categorization response (power across EEG spectrum) was obtained in 5-year-old children but was not right lateralized
Lochy et al. (2020) [80]	N = 52 Mean: 5.56; Range = 5.01–5.98	Fast Periodic Visual Stimulation	Signal to noise response (SNR) spectra	1. In the individual face discrimination paradigm, the response was mainly located on the right lateral site 2. In the generic face categorization paradigm, the response was bilateral and spread over dorsal, lateral, and posterior sites 3. Inversion of faces decreased the individual discrimination response
Meaux et al. (2014) [81]	N = 26 Mean: 5.08 (0.37) (Also 6–8, 8–10 year olds)	Faces (Batty & Taylor, 2003)	Amplitude and latency of P1, N170 and P2 ERP components	P1 amplitude was larger over the right hemisphere than left in 4–6 year old children Change across age: 1. P1 amplitude but not latency decreases 4 to 10 years of age 2. N170 latency decreases from 4 to 10 years of age 3. N170 amplitude decreases from 4 to 8 years of age 4. P2 latency and amplitude decrease from 4 to 10 years of age Change across gender: 1. No effect of gender on P1, N120 and P2 amplitude or latency
Melinder et al. (2010) [82]	N = 12 Mean: 5.28 (0.27)	Pictures of children, adults and elderly adults	Amplitude and latency of P1, N170 and P2 ERP components	1. Viewing faces of children elicited the smallest P1, P2 and largest N170 amplitudes and these differed from elderly faces but not from adult faces 2. Inverted faces elicited larger P1 and P2 amplitudes and longer P1 and P2 latencies than upright faces Change across age: 1. Longer P1 latencies and larger P1 latency differences between upright and inverted faces in 5-year old children compared to adults 2. Children’s N2 amplitudes significantly differed between upright and inverted faces whereas adults’ did not 3. Adults showed faster N170 latencies to upright than inverted faces while children did not
Carver et al. (2003) [83]	N = 42 Range: 2–3.75 (n = 14) 3.75–4.5 (n = 14) (Also 1.5–2 year olds)	Mother vs stranger	Amplitude and latency of Nc and P400 ERP components	Change across age: 1. Children of all ages showed larger amplitude Nc and P400 components to unfamiliar than familiar toy stimuli 2. Children between 18 and 24 months showed greater Nc amplitude to the mother’s face than to a stranger’s face while children between 45 and 54 months showed the opposite. Children between 24 and 45 months did not show differential responses to a mother’s and a stranger’s faces
Emotional stimuli processing–faces				
Batty and Taylor (2006) [84]	N = 82 Mean: 4.8 (n = 13) (Also 6–7, 8–9, 10–11, 12–13 and 14–15 year olds)	Neutral vs emotional faces	Amplitude and latency of P1 and N170 ERP components	1. No effect of emotion on P1 amplitude 2. P1 evoked by fearful faces had longer latency than neutral, happy and surprised faces and 3. P1 evoked by happy faces has shorter latency than that evoked by disgust, fear and sadness 4. Happy faces evoked a smaller negative activity in fronto-central sites than disgust, fear or sadness Change across age#: 1. P1 amplitude and latency decreased with increasing age 2. N170 amplitude decreased from 4–5 years of age until 12–13 years and then increased at 14–15 till adulthood 3. N170 latency decreased with age from 7–10 years 4. The mean fronto-central amplitude, which is positive in adults, was negative in children 5. P1 and N170 amplitude was larger over the right hemisphere sites than left Change across gender: 1. Boys had longer P1 latency than girls for all emotions except sad and angry faces 2. Larger N170 amplitude for girls than boys in the right hemisphere
Vlamings et al. (2010) [85]	N = 20 Mean: 3.10 (Also 6–8 year old children)	Neutral vs fearful faces	Amplitude and latency of P1 and N170 ERP components	1. Higher spatial frequency (HSF) images (such as contours of the eyes, eyebrows, mouth, and so on) elicited effects in ERP components while LSF did not 2. Higher P1 amplitudes for fearful compared to neutral faces 3. Higher N170 amplitudes for neutral compared to fearful faces Change across age: P1 amplitude decreased with age
Jiang et al. (2017) [86]	N = 27 Range: 4.5–5.5	Neutral vs emotional faces	Alpha, beta delta, gamma, theta band power	Compared with neutral stimuli, negative stimuli induced greater theta event-related synchronisation in children for whom negative emotional content was associated with improved cognitive efficiency
Emotional stimuli processing–non-faces				
Theall-Honey and Schmidt (2006) [87]	N = 36 Mean: 4.5 High shy (n = 18) Low shy (n = 18)	Video clips to induce sadness, anger, happiness, and fear	Alpha (6–8 Hz) power	1. Greater relative right central alpha power at rest in shy compared with non-shy children. 2. Greatest relative right anterior alpha asymmetry in response to the affective fear condition in shy children Change across gender: Greater relative right mid-frontal alpha power activation during the sad, happy, and fear video clips in shy females than males who displayed greater relative left mid- frontal alpha power activation
Cheng et al. (2014) [88]	N = 57 Mean = 5.1 (0.57) (n = 18) (Also 6–7, 8–9 year-olds and 23 year old adults)	Dynamic visual stimuli of limbs in painful vs non-painful situations	1. Amplitude of Early Automatic Component (EAC) and Late Positive Potential (LPP) 2. Mu suppression	The Pain relative to No-pain condition elicited a more positive-going EAC response. Change across age: 1. Between 4–9 years age, the difference in the EAC amplitudes between Pain and No-pain decreased 2. Difference wave (Pain minus No-pain condition) of LPP increased with age 3. Stronger mu suppression in children with no differentiation between painful and non-painful stimuli Change across gender: No gender difference in the EAC and LPP response
Hua et al. (2014) [89]	N = 20 Mean: 5.08 (0.64)	Pleasant, unpleasant and neutral pictures	Amplitude of Late Positive Potential (LPP)	Larger LPP amplitudes to unpleasant and pleasant than neutral pictures in the posterior region in the early time window (300-700ms post stimulus onset), in the central region during the middle (700-1500ms post stimulus onset) and late time window (1500-3000ms post stimulus onset) and in the anterior region during the late time window
Hua et al. (2015) [90]	N = 20 Mean: 4.97 (0.67)	Negative vs neutral interpretation of pictures	Amplitude of Late Positive Potential (LPP)	1. LPP amplitudes following neutral interpretations were lower as compared to negative interpretations in children as young as 4 years old 2. LPP amplitudes were maximal in the early time window (400-1000ms post stimulus onset) for the posterior region, but maximal in the middle (1000-2000ms post stimulus onset) and late time windows (2000-3000ms post stimulus onset) for the central and anterior regions Change across age: No effect of age on LPP amplitude
Mai et al. (2011) [91]	N = 13 Mean = 4.43–5.34	Prize guessing game	Amplitude and latency of Feedback-Related Negativity (FRN) and P1. Amplitude of Positive Slow Wave (PSW)	1. P1 had larger amplitude and longer latency for good prizes compared to bad prizes 2. PSW amplitude was larger for good prizes than bad prizes in the right central parietal area 3. FRN had no differences between good and bad prizes

A total of 48 of 51 studies were conducted in high-income countries with the majority (34/48) being from USA. Many studies (19/48) did not specify the ethnicity of their participants and in those that did, a lack of diversity was evident, with the average proportion of Caucasians being 79.2% (SD: 16.4%) (S3 Table). Three studies were published from China, representing an upper-middle-income country. None of the studies identified in this review were conducted in LMICs and only six studies sampled children from low income families to analyse the impact of socioeconomic status (SES) on brain activity. All of the remaining studies that mention the SES of their participants (15/48 did not specify it) report that they are from middle to high income families with high levels of education (S3 Table).

#### EEG data collection and pre-processing procedures

There was a large diversity in the equipment used to collect EEG data in these studies (S4 Table). The number of electrodes ranged from 5 to 128 (low to high density), with most studies using the 10/20 array. Sampling rate ranged from 100 to 2000 Hz. Data pre-processing techniques used in these studies also differed greatly making it hard to compare findings across studies (S5 Table). The data was either band-pass filtered from 0.1 or 1 to 30 or 40 Hz, or a stepped approach was taken applying a band-pass filter of 0.1 to 100 Hz first and then followed by a low-pass filter at 30 Hz (typically for ERPs). Only 3 studies reported filtering for line noise at 50 or 60 Hz. Most often the vertex (Cz electrode) was used as a reference during recording (27/51), while use of mastoids as references was less frequent (18/51). The data was then re-referenced offline to the average of all electrodes in 28 studies, of which 15 had a relatively low electrode density (<60 electrodes). To further clean the data, thresholds were used for automated identification of artefacts, for example, a peak-to-peak criterion of greater than 100 μV and 200 μV for eye blinks or movements and gross motor movements, respectively. This was typically followed by visual inspection of the data and subsequent removal of artefacts in almost all studies. EEG data was segmented and, depending on the task used in the study, corrected for baseline, which ranged from 50 to 600ms (with the majority of the studies using 100 to 200ms) before stimulus onset. In ERP studies, the time-window analysed depended on the ERP components of interest. However, the range of the time-window differed between studies examining the same component, as some studies defined time-windows *a-priori* while others used a data-driven approach based on grand average ERP of their sample. Similar differences between studies were found for the definition of regions or electrodes of interest, which could either be predetermined or automatically identified with for example Principal Component Analysis (PCA).

#### Assessment of risk of bias

**Fig 2:**
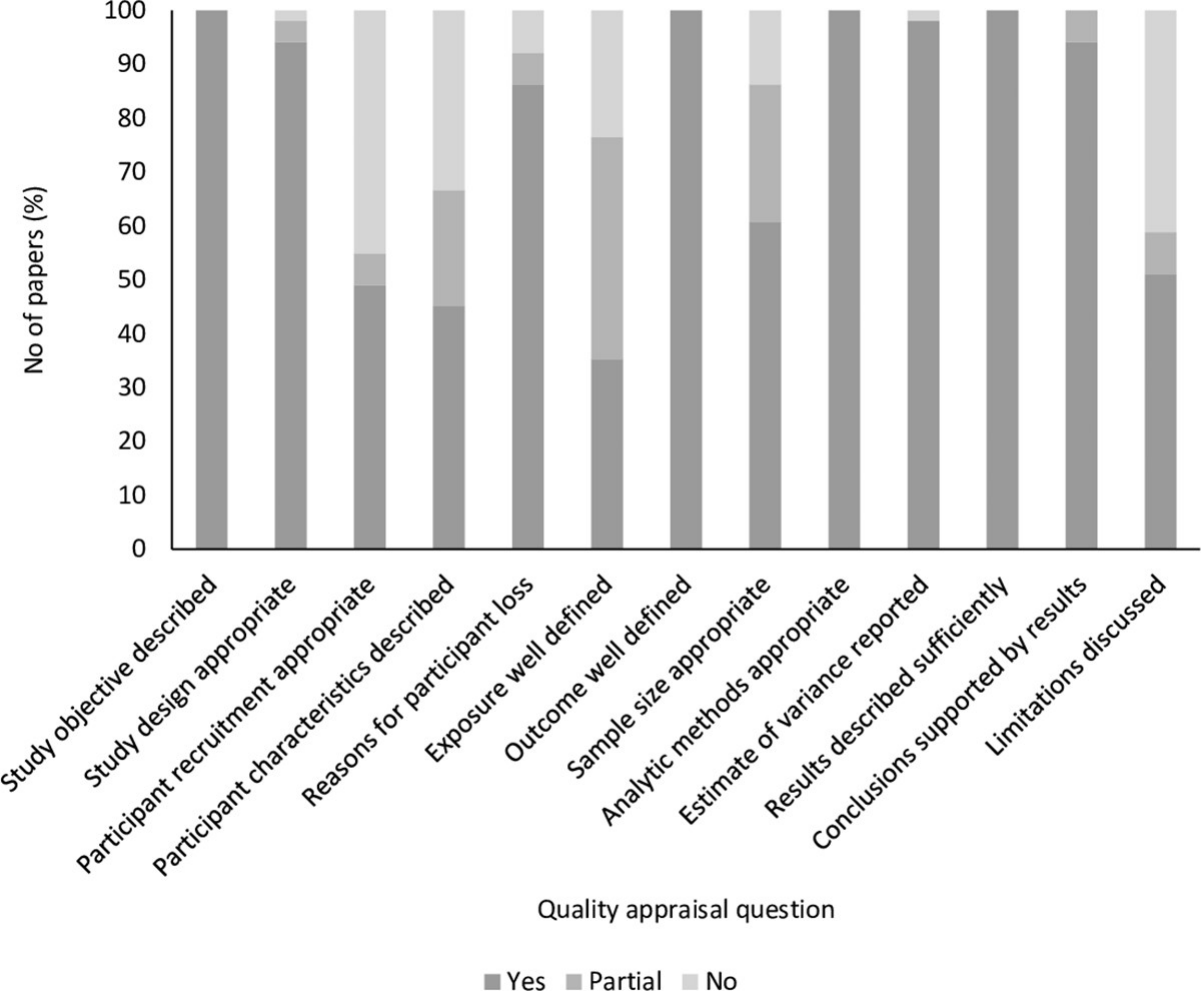
Assessment of risk of bias—percentage of papers scoring ‘Yes’, ‘Partial’ and ‘No’ in response to quality appraisal questions.

The results of the quality appraisal of all included studies are summarised in Fig 2 (see S2 Table for questions). Methodological quality of most studies was high (93–100% studies scored ‘yes’) when appraised for their research question, study design, definition of outcome (EEG) measures and reporting of analytic methods, results and conclusions. However, only 61% of studies included an adequate number of participants (N ≥ 20) in each analysed group. About half of the studies received ‘partial’ or ‘no’ scores on criteria assessing the method of recruitment and description of participants such as sociodemographic details. Interestingly, a large number of the studies either did not at all (24%), or did only partially (41%), report on the method employed to assess whether participating children were developing typically, for example by assessing children’s development using validated scales such as Mullen’s Scale of Early Learning [92] and Wechsler Intelligence Scale for Children [93]. Most studies mentioned the number of participants excluded from their analysis, along with sufficient details on the reasons for exclusion, however, they did not disaggregate this loss of participants across their analysis groups like age. 41% of the included studies did not report limitations of their study. Those that did highlighted technical limitations in the EEG equipment or data collection techniques, challenges of interpreting child performance on their tasks, small sample sizes and the use of homogenous populations.

### EEG signatures of cognitive abilities

The data on age range and sample size of participants, tasks used, EEG measure analysed and significant findings extracted from the 51 included studies are summarized in Table 1 and described below. Most commonly reported measures were the EEG power spectrum or the amplitude and latency of ERPs. The former refers to relative fraction of the power spectrum of defined frequency bands (e.g. 6–10 Hz for alpha power). Identification of ERP components depends on the task being used (see S2 Fig for a sample trace in response to faces) and these are defined in two ways: a) based on the order of positive and negative deflections, for instance P1 is the first positive deflection or b) based on the latency with which they occur, such as N170, which is a negative peak around 170ms after stimulus onset. We categorise the domains of cognitive and social development reported in these studies into executive function, selective auditory attention, learning and memory, and processing of faces and emotional stimuli.

#### Executive function (EF): Visual attention, working memory and inhibitory control

**Fig 3:**
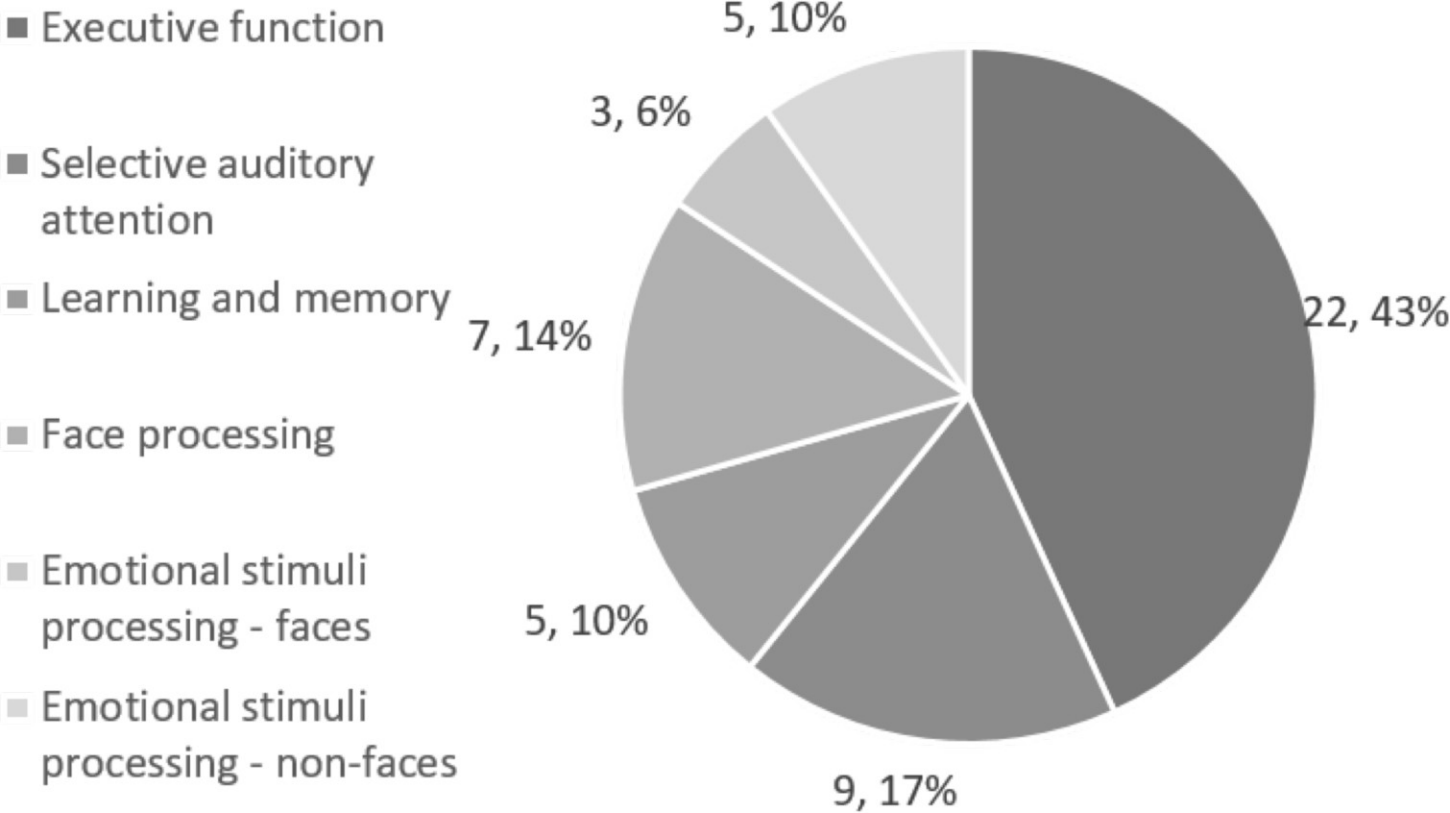
Distribution of papers across domains of cognitive and socio-emotional development.

Fig 3 shows that 22 of 51 studies identified in this review assessed executive function, using multiple tasks: changes in EEG spectral power during Stroop and Yes-No tasks (8/22) was most commonly studied, followed by ERPs in Go/No-Go (6), Flanker (3) and set-shifting tasks (3) like Dimensional Change Card Sort (DCCS) or Ocean Sort tasks (tasks described below). The results in this section begin with studies measuring ERPs followed by spectral power, and start with the most commonly used tasks.

*Go/No-Go task*. Six studies used the Go/No-Go task for response inhibition in which participants have to respond in the majority of the task trials (Go condition), but withhold their response when a particular stimulus appears (No-Go condition). Four of these studies were conducted with 5-year old children and the other two included younger age groups. Five studies reported on the amplitude and latency of the frontal negative (specifically including N2) ERP component while two reported on the positive (P1 and P3) ERP components. One study demonstrated left lateralisation of frontal negativity in both Go and No-Go trials [43] and another reported that relative to Go trials, No-Go trials elicited longer N2 latencies [43,44]. NoGo trials also elicited larger negative (N2) and positive (P3b) amplitudes compared to Go trials [43,45,47]. A study used source localisation analytic techniques to demonstrate an asymmetrical pattern of scalp lateralization of N2: right-lateralized in No-go trials and left-lateralized in Go trials [42]. One study investigated change across gender and found no significant association [47]. Brooker et al found no change across age from 3.5-4-year olds but found that the ability of Error-Related Negativity (ERN) at 3-years age to predict ERN at 4-years age was dependent on SES [46].

*Flanker task*. In the Flanker task participants are instructed to respond to a central relevant stimulus which is ‘flanked’ on either side by irrelevant stimuli that can either be congruent or incongruent with the central stimulus, and is primarily a measure of response inhibition. All three studies using this task in children aged 4.3–5.5 years reported that the negative amplitude of the N components over frontal electrodes (N1 and N2 ERP components) were modulated by the congruency of the trials [48–50]. Additionally, the amplitude of the frontal positive ERP component, P3 was larger and with a longer latency in incongruent compared to congruent trials [48]. The P3 amplitude was found to be negatively correlated with performance on the task [50]. Rueda and colleagues demonstrated age-related differences in both negative and positive ERP components (N1, N2 and P3) between children and adults: N1 and N2 amplitudes and N1, N2 and P3 latencies decreased significantly with age [48]. They also demonstrated a change in lateralisation of the larger P3 amplitude and longer latency from the right to left hemisphere as age increased from 4-years age to adulthood. One study investigated the impact of SES on N2 and P3 amplitudes and found no significant association [50].

*Set-shifting tasks*. Three studies assessed cognitive flexibility through the use of set-shifting tasks like DCCS or Ocean Sort tasks in which participants are expected to start the task by sorting objects based on a particular dimension (like colour) and switch to another dimension (like shape) in the middle of the task. One reported larger P3 amplitude and shorter P2 latency at the right fronto-central and left parieto-central electrode clusters respectively in switch than stay trails in 5-year olds [55]. Another showed that fronto-central N2 amplitude, but not latency was smaller in 3.5-year old children who switched flexibly between sets [52]. The third study found that medium-to-high frontal alpha power moderated the positive relationship between child performance on EF and SFT tasks [53].

*Stroop/Yes-No task*. Eight papers used the Stroop task, some in conjunction with the Yes-No task, to index executive function in which participants have to remember to give a response which is opposite to the stimulus which they process (for example, saying ‘day’ when they see a black card with a moon, or saying ‘no’ when the experimenter nods their head). All eight studies measured power changes in the alpha frequency band in frontal regions, mostly defined as 6–9 Hz and two of them also measured intra-hemispheric connectivity using inter-channel coherence. Increased baseline to task alpha power in the left medial frontal region, along with language and temperament, were found to predict the performance of 3.5- and 4-year olds on the Stroop task [56,58,59] and, these factors, together with maternal education, also predicted child performance on the Hand Game task which follows principles similar to the Stroop and Yes-No tasks [60]. This group also demonstrated that alpha power increased with increasing executive demands in 4-year old children [62]. A study showed that high performers in EF tasks had increased medial frontal alpha power as compared to low performers in 3.5–4.5-year olds, and this effect was mediated by shyness [61]. Moreover, increased alpha power during EF tasks were demonstrated to be dependent on age decreasing from infancy [57] and 3.5 years [58] to 4.5 years. Baseline to task increase in alpha power and decrease in coherence across electrode pairs, also became more localised across this age range moving from being observed over the entire scalp in infants to more localised scalp regions in the older children [57,58].

One study investigated the impact of gender on alpha power during EF tasks in 4-year olds, and found that girls exhibited higher overall power which was more localised, when compared to boys [62]. Interestingly, another study using the Crayon/Marker Delay task to assess inhibitory control in younger children (2-year olds) also demonstrated the association between frontal alpha power and child performance. They however did not find any differences based on gender [51]. Finally, one study used a battery of six EF tasks including Stroop, Go-NoGo and Working Memory Span to derive a single EF performance score, in 4-year olds and demonstrated that frontal alpha power was unrelated to child performance while a decrease from baseline to task coherence was associated with performance [63].

In summary, 18 studies assessed EF using a variety of tasks and reported on varied metrics including alpha power and the amplitude and latency of ERP components showing significant associations with behavioural performance. Most notably, in 3.5–4.5-year old children, an increase in alpha power from baseline to task was associated with EF as measured by Stroop and Yes-No tasks [57,58,61,62].

#### Selective auditory attention

Nine studies measured selective attention using auditory tasks (See Fig 3) in which stimuli such as stories or environmental sounds were presented from two audio sources. Children had to selectively attend to the stimulus from one channel and inhibit their attention from the other. Studies with 5-yr old children showed a broad positivity across all electrode sites that peaked around 100ms after stimulus onset, rather than the positive-negative-positive (P1-N1 complex) ERP waveform characteristic of adults [65,67,69], which does not emerge until early adolescence [69]. While pre-schoolers did not have the ability to differentially attend to stories played in two channels [68], they could selectively attend to environmental sounds [67] as evidenced by the absence [68] and presence [67] of a negative oscillation in the ERP waveform respectively.

Larger P3a amplitude in the fontal and central electrodes to novel attention-catching sounds was found to be associated with temperamental traits like higher effortful control, higher negative emotionality (such as sadness and fearfulness and feelings of discomfort) and more synchronous parent-child interaction in 2-year old children [66]. A larger mean amplitude difference between 100-200ms post stimulus onset in response to attended as compared to unattended stimuli (attention effect) in anterior and central electrode locations was also found to be correlated with superior non-verbal IQ scores in older 4-year old children recruited from low-income households [70]. Interestingly, this group subsequently showed that children from low SES were delayed by one year in development of this attention effect as compared to those from high SES as demonstrated by a 1-year follow up of the low SES sub-group [71]. Larger positive amplitudes were also elicited by distracting sounds in children with lower SES [72].

#### Learning and memory

In order to evaluate neural bases of learning and memory, five studies identified in this section compared ERP components in response to familiar or previously viewed objects compared to novel ones. However, the paradigms differed in duration with two investigating immediate recall, one each investigating recall delayed by 5-minutes, one or seven days. ERPs in response to correctly versus incorrectly recalled images were found to be more positive i.e. they had less pronounced negativity, in 2.5-, 3- and 4-year old children [14,73,76]. A study showed that the amplitude of the positive slow wave (PSW) in frontocentral sites decreased with increasing recall of temporal order in which items were presented to 3-4-year old children and PSW amplitude and latency decreased across these ages [73]. Yet another study demonstrated age-related changes in a mid-latency negative component, Nc, and PSW in response to items recalled with contextual details, which increased between 3 and 4 years, and items recalled without contextual details, which were greatest in 5- year-old children. However the location of this effect differed between the age groups, moving from frontal parietal leads in 3-year olds to the left hemisphere leads in 4 year olds [74]. However, another study found no association between Nc in the frontal and central electrodes and age when comparing children younger than 5.8-years with older ones [75].

#### Face processing

Faces represent a very important visual stimulus, particularly in social development. This review identified seven studies that measured neural specialisation towards processing of facial information, and these included tasks in which images of upright and inverted, familiar and unfamiliar, and scrambled faces were presented to participants. None of these studies investigated the impact of socio-economic status on face processing. One group reported the response of the power spectrum to faces in 5-year old children [79,80]. All other studies reported ERP components and the results for this section are segregated based on these ERP components.

*P1*. P1 in the occipital region is a commonly studied ERP component in children that occurs early during the visual processing of faces, or indeed any complex visual stimulus. It is thought to reflect early stage cortical processing of patterns and objects. Its amplitude was larger in response to older than younger faces [78,82] and increased across childhood [77,81,84]. P1 amplitude was also larger on the midline compared to right and left hemispheres in 3.5-year olds [78], but was right lateralised in 4-6-year olds [81]. P1 latency to inverted faces decreased with age from early childhood to adolescence and adulthood in three studies [77,78,84], but not in a fourth which had a more limited age range (5-10-year old children) [81]; and was found to be shorter for inverted compared to upright faces in 3.5-year old [78] and with the reverse being demonstrated in 5-year old children [82].

*N170*. The N170 is an ERP component recorded over temporal lobe channels that was measured as a strong negative response for faces compared to other objects in adults. From the second year of life, a strong negative inflection occurs over temporal sites in response to faces and its latency gradually decreases to be adult like in late adolescence. Given the continuity in functional properties this is referred to as the N170 through childhood, despite the longer latencies at which it occurs [94,95]. Its amplitude was larger, with shorter latencies, to eyes than faces, in 4-5-year olds and larger over the right hemisphere in all age groups [77]. N170 amplitude was also larger to face than non-face images and to adult than infant faces [77,78]. Melinder and colleagues (2010) showed that viewing faces of children elicited the largest N170 amplitude compared to viewing faces of older people [82]. N170 amplitude decreased from 4 to 8 years of age in one study [81], and from 4–5 years until 12–13 years of age in another [84]. Taylor and colleagues [77] found this pattern of change only in response to eyes. Lateralisation of the N170 response to inverted and upright faces to the left hemisphere was stronger in 4-5-year olds as opposed to adolescents and adults [77] and stronger in the right hemisphere for girls than boys [84]. N170 latency in response to faces decreased with age from 4–10 years [81] and also when 4-5-year old children were compared with adults [77,82,84]. N170 latency and amplitude showed no difference based on gender in younger children (5-10-year olds) [77,81] but was found to be faster and larger in girls than boys in older age groups [77].

*Other ERP components*. Other than P1 and N170, a few studies reported significant findings in relation to face processing in other ERP components, namely the P2 and the P400 in the occipital region. Larger P2 amplitudes with longer latencies for inverted than upright faces have been shown in 5-year old children [82] and in addition, P2 amplitude and latency in response to faces decrease from 4 to 10 years of age [81]. P400 response is larger to inverted than upright faces in 3.5-year old children [78] and to familiar compared to unfamiliar toys in 2-5-year olds [83].

To summarise, most studies assessing children’s neural processing of faces measured the P1 and N170 ERP components. The majority of these studies reported on changes in these components across age. For instance, P1 latency to inverted faces and the N170 amplitude and latency in response to faces were demonstrated to decrease with age [77,78,81,82,84].

#### Emotional stimuli processing–faces

This review identified three studies that investigated the response of face processing ERP components described above to neutral, positive (happy, surprised) or negative (fearful, sad) emotional facial expressions. One study found that the P1 amplitude was higher when observing fearful compared to neutral faces in 3-year olds [85] while another study found no effect of emotion in 4-year olds [84]. The P1 latency was shown to be sensitive to variations in emotional expression in perceived faces in children approximately 5-years old, such that the P1 occurred early in response to neutral and positive emotions, and later for negative ones like fear and disgust, and to gender of participants, with longer latencies to all emotions except sad and angry faces in boys than girls [84]. The impact of emotions on N170 amplitude were demonstrated to be larger for neutral compared to fearful faces in 3-year olds [85] but no effect of emotion on the N170 was found in a study of participants ranging in age from 4–15 years [84]. Finally, a recent study of spectral power found evidence of higher synchronization in the theta band in response to negative emotional expressions in the subset of children for whom negative emotional content was associated with improved cognitive efficiency [86].

#### Emotional stimuli processing–non-faces

Of the five studies examining the processing of emotional stimuli other than faces such as cute compared to fierce animals or disaster pictures, the EEG measure studied most often (3/5 studies) was the late positive potential (LPP) in posterior, central and frontal regions. LPP was found to be larger to negative and unpleasant stimuli than neutral and pleasant ones [88–90]. Changes in LPP amplitude across development remain to be established. One study reported no effect of age on LPP amplitude in response to negative or neutral interpretations of images in 4 to 5-year olds [90]. On the other hand, using images depicting painful and non-painful situations, LPP amplitude has been demonstrated to increase from 4 to 9 years and adulthood, with no differences found between boys and girls [88]. One study used a unique prize guessing game in which children attached a value of ‘good’ or ‘bad’ to objects and found larger amplitude and longer latency for the P1 in the parietal and the positive slow wave (PSW) in the central parietal areas, but not in feedback-related negativity (FRN) elicited in response to ‘good’ as compared to ‘bad’ objects [91]. The final study in this category was unique in that it analysed alpha (6–8 Hz) power in response to video-clips designed to induce emotions such as sadness, happiness, anger and fear in shy as compared to non-shy children, and found greater frontal asymmetry in shy children in response to fear [87].

## Discussion

This systematic review presents a comprehensive synthesis of studies conducted over the last three decades that have used electroencephalography to measure neural correlates of cognitive and social development in 2-5-year old children. Even across the target age range of this review, participants were found to be unequally distributed with more studies focusing on older 4-5-year old children, an easier age group to collect EEG data from, with significantly lower attrition rates due to cap refusal and less movement artefacts, when compared to younger toddlers [19]. Optimising protocols to engage young children while applying EEG electrodes to reduce attrition rates, and designing age-appropriate tasks, while at the same time keeping gross motor movements to a minimum, presents a challenge that is yet to be completely overcome in this field of study [19].

The heterogeneity of the studies identified in this review is evident from an analysis of the equipment and methods used to collect EEG data and subsequently pre-process and analyse it. Studies differ with respect to the tasks that they use to measure the same cognitive domains and even within the same task used at the same age, use of different stimuli or analysis of different EEG metrics often limits the ability to synthesise their findings and limits their reproducibility [52,55]. A future way forward is to develop a common EEG platform with associated software that allows for compatible data collections across multiple sites and populations [96–98].

The assessment of risk of bias conducted in this review demonstrates the strength of included studies in reporting methods and results in relation to outcomes of EEG measures, but also revealed that studies were limited in their reporting of participant details such as recruitment criteria, sociodemographic profile and methods employed to test whether children included in the study were developing appropriate to their age. This field of research would additionally benefit from reporting more details of their study population, and making greater efforts to increase their diversity to allow for generalisability and replicability of their results [99].

Another common limitation of these studies, often acknowledged by authors themselves, is the small sample size, often amplified by the loss of participants either during data collection or analysis. This limitation is, of course, closely linked to the challenge of collecting data on young children as discussed above. This challenge of extensive loss of data highlight the need to take these high attrition rates into account while sampling and use data processing methods that generate robust EEG signatures derived from ‘imperfect’ datasets which would result in more studies with larger sample sizes. Again, harmonization of technology and methods can help with this issue [96–98].

### Emerging evidence for neural biomarkers of cognitive and social development

Bearing in mind the heterogeneity of the identified studies, this review provides a synthesis of some key insights into neural correlates of cognitive and social development in preschool children. Two studies identified in this review have demonstrated that No-Go N2 amplitudes are larger than Go trials in 2–5 year old children [44,45], a result that has also been found in another systematic review and meta-analysis on 2-12-year old children [31]. Four studies, identified in this review, conducted on 3.5–4.5-year old children demonstrated an increase in alpha power from baseline to task in the medial frontal region as being associated with executive function as measured by Stroop and Yes-No tasks [57,58,61,62]. This finding is unsurprising considering that research has consistently shown significant frontal activation during EF tasks in typically developing infants and children [100–102]. In three ERP studies on 3.5–5 year olds investigating selective attention using auditory tasks, a broad positivity has been observed 100ms post stimulus presentation as opposed to the distinct P1-N1 complex which is routinely seen in adults [65,67,69].

The studies described in this review have highlighted the importance of the mid-latency negative component (Nc) and the positive slow wave (PSW) in tasks involving learning and memory. The amplitude of PSW is greater and Nc is smaller (has less pronounced negativity so it is also more positive) in correctly compared to incorrectly recalled items [14,76]. Similar to this finding, the Nc component has been demonstrated in infants to be larger in amplitude to images that capture infants’ attention the most for instance favourite over novel toys and mother’s face over a stranger [103].

Consistent with previously published reviews, the P1 and N170 ERP components have emerged as the most commonly studied responses during processing faces. Taylor and colleagues demonstrated shorter latencies for the P1 in response to upright compared with inverted faces in 4-15-year old children [95]. This is consistent with another study included in this review in 5-year olds [82], while longer P1 latencies to upright than inverted faces were demonstrated in a study with younger participants (3.5-year olds) [78]. This seems to imply that an important developmental change occurs between these ages and warrants further investigation. One study found that the N170 amplitude was larger (more negative) to facial than non-facial stimuli in 4–5 year old children [77]. Many published studies on both children and adults concur with this finding, suggesting that this response can also be interpreted as the N170 being face-sensitive rather than face-selective, such that it is larger to objects of visual expertise, with faces being one of the objects for which most people are experts [95,104]. The N170 was found to be larger in response to fearful than neutral faces in 3-year olds [85]. This is in contrast to a study in 7–13 year old children [105], casting light on the complexity of the changes in this measure across childhood and adolescence.

A total of three studies included in this review examined the late positive potential (LPP) elicited in response to stimuli with emotional valence. More specifically, the LPP was shown to be larger in response to negative and unpleasant stimuli than positive and neutral ones in 4-5-year old children [89,90]. This finding is consistent with results from older 5-7-year old children [106]. Interestingly, the LPP measured in middle childhood has been shown to be predictive of later emotional regulation capacity [107], suggesting its importance as a potential neurophysiological marker of typical emotional development in preschool children.

### Changes in EEG measures across development, gender and socioeconomic status

The secondary aims of this review included elucidating changes in EEG measures across age, gender and socioeconomic status. Despite a narrow age-range included in this review, some subtle differences in EEG measures of cognitive and social development across ages have been identified. In particular, the reduction in alpha power in the medial frontal region during executive function tasks increases with age between infancy and 4.5 years [58,61]. Another observation of the review is that N170 amplitude and latency and P1 latency in response to faces decrease with increasing age [77,81,82], consistent with prior reports [95,108]. The decrease across age in P1 and N1 latency and N2 amplitude in response to the Flanker task demonstrated in a study in this review [48] has also recently emerged as a finding of a study conducted after this review search investigating developmental changes of selective attention [109].

Only 8 of the 51 included studies either disaggregated their data by gender or included gender as a variable in the analysis. This is intriguing given there is some evidence that neurophysiological processes differ based on gender. For instance, it has been found that girls have a higher level of synchronisation for all frequency bands than boys during resting state at 5–7 years [110]. This points to a need for a concerted effort by the EEG research community to further understand the scale and nature of brain differences between genders.

Only six studies examined the impact of SES of which five demonstrated its effect on EEG metrics of Go/NoGo and selective auditory attention with larger amplitudes of positive and negative ERP components in children from lower SES [47,71,72]. This larger amplitude manifested as a delay in the development of selective auditory attention in low-SES children when compared to their high-SES peers [71]. However, one study found no impact of SES on attention and inhibitory control as measured by the Flanker Task [50]. The importance of SES highlighted by these few and recently published studies suggests that more research needs to be conducted to investigate risk factors that are known to influence trajectories of neural development and functioning in children [13]. There is some literature, outside the scope of this review, on the impact of low SES on resting state and task-based neural activity using fMRI and EEG; this research shows delayed maturation of both neuronal markers of basic sensory processing, as well as higher order processes such as brain oscillations in frontal regions that index inhibitory control [38,111,112]. However, the majority of this research has focused either on infants or older children and needs to be expanded to include the crucial preschool years. Of note, while some neurophysiological studies have begun emerging from LMICs, this review did not identify any studies using cognitive or social tasks within the target age-range that were conducted in LMICs, highlighting a large research gap.

### Limitations of this review

While this review provides some unique insights into the state of developmental EEG research in the context of preschool children, one of its key limitations lies in the relatively narrow age range of 2–5 years, which might not be sufficient to capture developmental changes in some neural markers of emerging cognitive domains. However, we took the view that focusing on this age range brings to light the limited research in this age group compared to the large amount of research done in younger and older children. A second limitation of this review is the focus only on EEG studies assessing cognitive and social domains of development. A review of EEG studies assessing neural responses related to the sensory, language and motor domains were defined as being outside of the scope of this study, yet such functions are of course integral to healthy growth and development of children. A third limitation relates to publication bias as a) only studies published in English language were included in this review, b) only studies with significant results are likely to have been successfully published in peer reviewed journals and, c) within these studies, results of exploratory analyses not attaining significance might not have been reported.

### Implications and recommendations for future research

In order to realise the potential that EEG has to be used at scale to measure neurocognitive development in low resource settings, which are home to a disproportionately large number of children at risk of sub-optimal development, there is an urgent need for this field of research to a) identify measures that are robust enough to offer a good signal-to-noise ratio even with lower quality portable EEG systems with lower density arrays, b) move away from the use of a small and homogenous samples to allow for greater generalisability of results, c) to standardise methods and establish best practices for task and stimulus presentation, EEG data collection and pre-processing techniques that are adopted by diverse research groups to allow comparison across studies, d) move outside of the highly controlled laboratory settings in which they are currently being conducted and into community settings or households, where such technology is likely to be implemented at scale. Previous EEG research has shed great light into neurophysiological markers of cognitive function and social-emotional processing in infants at risk of developmental delays (through studies on preterm infants), and disorders like ASD and ADHD (through studies on children at familial risk such as siblings of children diagnosed with these disorders) which have been demonstrated to be predictive of their later manifestation [28,113–117]. These findings reinforce the urgent need for more research into the patterns with which these neural processes develop in neurotypical children to enable early identification of those who are at risk of faltering in their development, intervention selection and monitoring of the effectiveness of these interventions [118].

## Supporting information

S1 Checklist EEG SR PRISMA 2009 checklist. (DOC)

S1 Fig Age range of participants in included studies. (TIFF)

S2 Fig Sample ERP traces–ERPs named based on a) the order of positive and negative deflections (e.g. P1 (highlighted in blue), N1) or b) the latency with which they occur (e.g. N170 (highlighted in red), P300).
(TIFF)

S1 Table Detailed search terms. (DOCX)

S2 Table Final quality appraisal form. (DOCX)

S3 Table Participant details of included studies. (DOCX)

S4 Table Data acquisition setup of included studies. (DOCX)

S5 Table Data pre-processing details of included studies. (DOCX)
