# Supplementary figures and images for: EEG signatures of cognitive and social development of preschool children–a systematic review
Source: PLoS One. 2021 Feb 19;16(2):e0247223. doi: 10.1371/journal.pone.0247223 (PMC7895403; doi:10.1371/journal.pone.0247223)

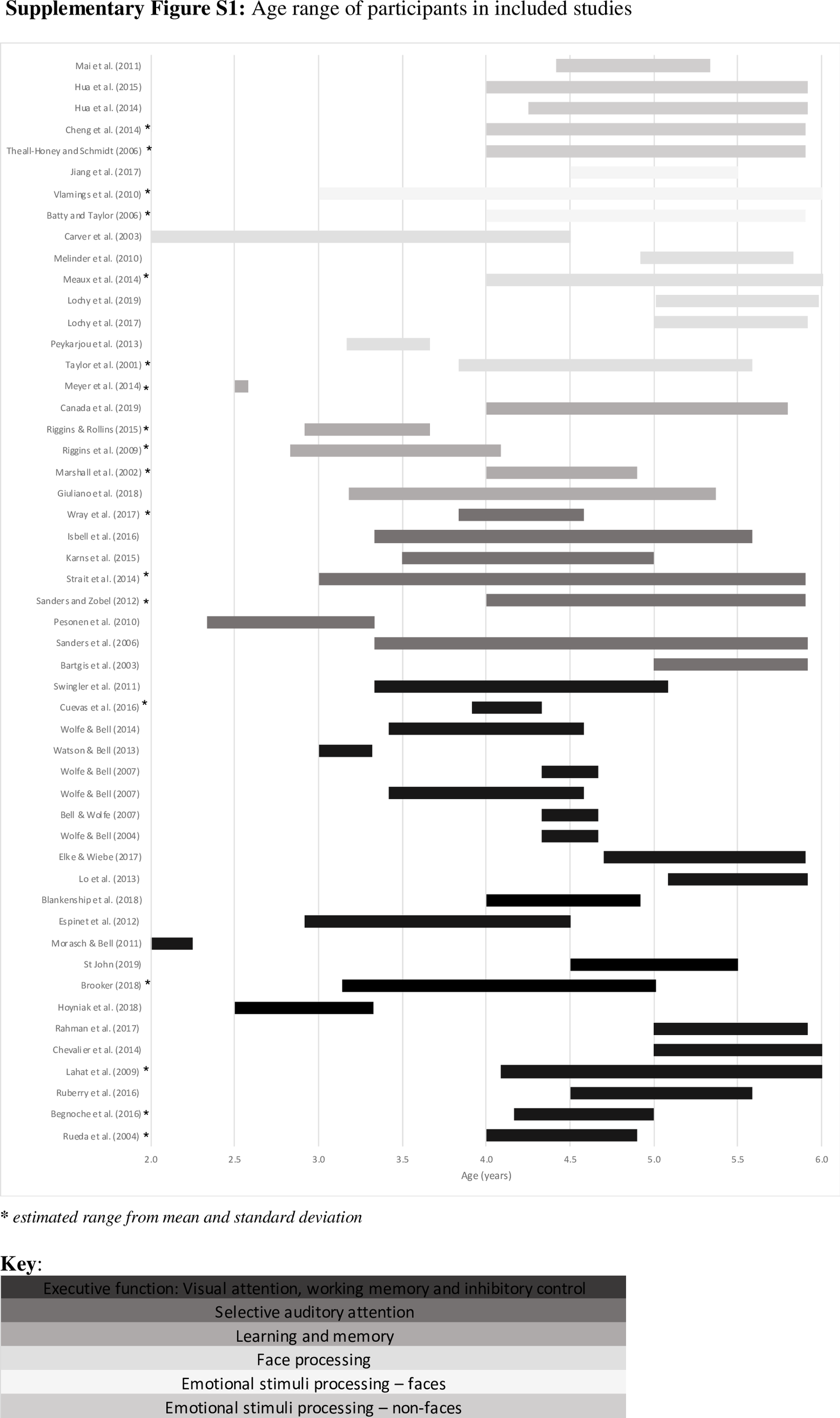

Supplement: S1 Fig — (TIFF) [file pone.0247223.s002.tiff]

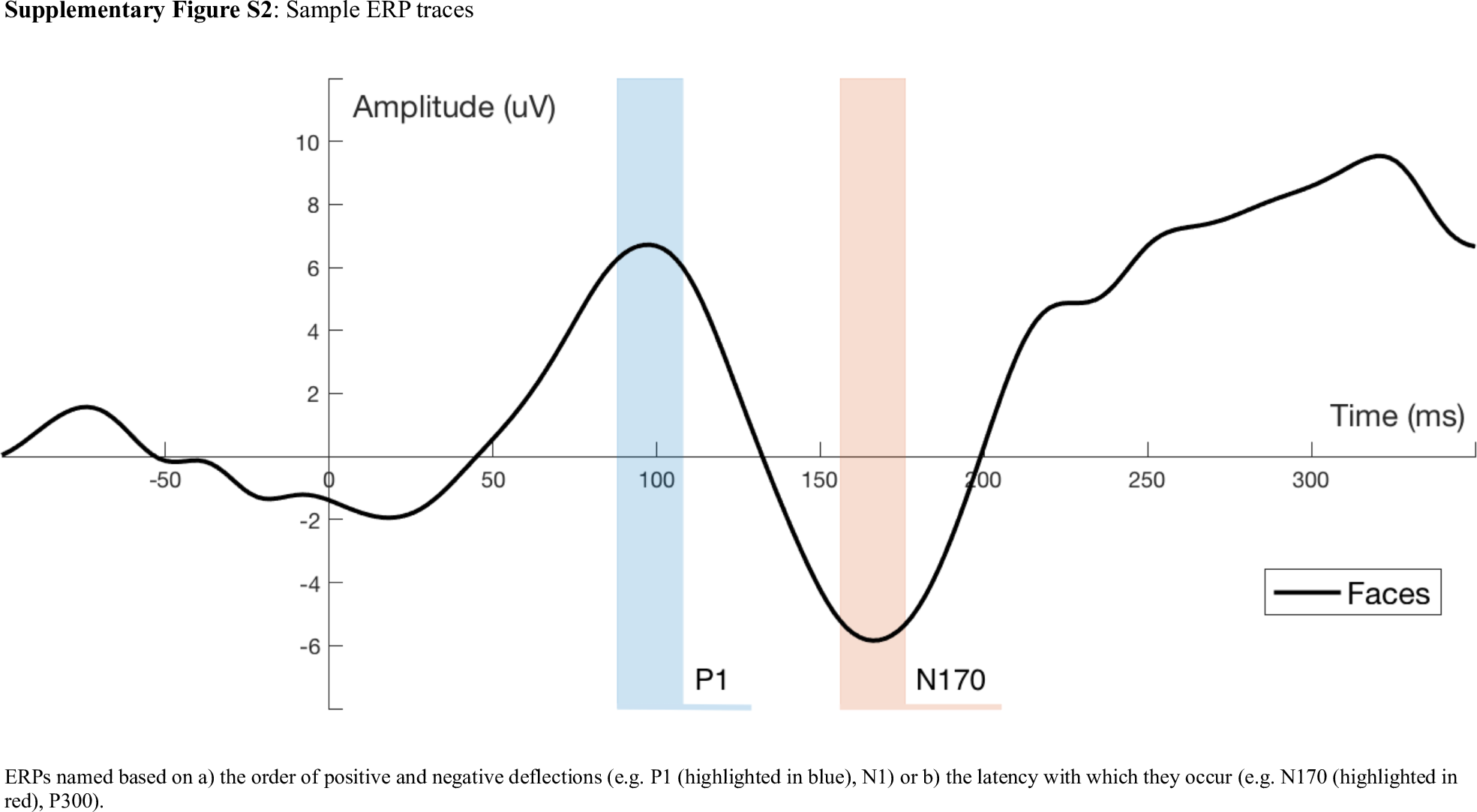

Supplement: S2 Fig — Sample ERP traces–ERPs named based on a) the order of positive and negative deflections (e.g. P1 (highlighted in blue), N1) or b) the latency with which they occur (e.g. N170 (highlighted in red), P300). (TIFF) [file pone.0247223.s003.tiff]
