# Supplementary material for: EEG signatures of cognitive and social development of preschool children–a systematic review
Source: PLoS One. 2021 Feb 19;16(2):e0247223. doi: 10.1371/journal.pone.0247223 (PMC7895403; doi:10.1371/journal.pone.0247223)
Supplement: S1 Table — (DOCX) [file pone.0247223.s004.docx]

**Supplementary Table S1**: Detailed search terms


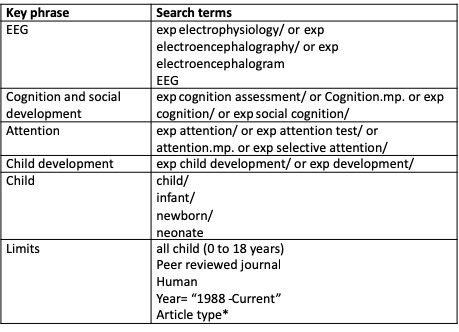


*(article or article in press or erratum or letter or classical article or clinical study or clinical trial, all or clinical trial, phase i or clinical trial, phase ii or clinical trial, phase iii or clinical trial, phase iv or clinical trial or comment or comparative study or controlled clinical trial or "corrected and republished article" or duplicate publication or evaluation studies or historical article or introductory journal article or journal article or multicenter study or observational study or overall or pragmatic clinical trial or published erratum or randomized controlled trial or technical report or twin study or validation studies or "0100 journal" or "0110 peer-reviewed journal")
