# Supplementary material for: EEG signatures of cognitive and social development of preschool children–a systematic review
Source: PLoS One. 2021 Feb 19;16(2):e0247223. doi: 10.1371/journal.pone.0247223 (PMC7895403; doi:10.1371/journal.pone.0247223)
Supplement: S2 Table — (DOCX) [file pone.0247223.s005.docx]

**Supplementary Table S2**: Final quality appraisal form

|  | Criteria | Yes | Partial | No |
| --- | --- | --- | --- | --- |
| 1 | Question / objective sufficiently described? |  |  |  |
| 2 | Study design evident and appropriate? |  |  |  |
| 3 | Method of subject selection described and appropriate? |  |  |  |
| 4 | Subject characteristics sufficiently described? |  |  |  |
| 5 | Reasons for loss of participants sufficiently described? * |  |  |  |
| 6 | Exposure measure(s) well defined? Means of assessment reported? |  |  |  |
| 7 | Outcome measure(s) well defined? Means of assessment reported? |  |  |  |
| 8 | Sample size appropriate? |  |  |  |
| 9 | Analytic methods described/justified and appropriate? |  |  |  |
| 10 | Some estimate of variance is reported for the main results? |  |  |  |
| 11 | Results reported in sufficient detail? |  |  |  |
| 12 | Conclusions supported by the results? |  |  |  |
| 13 | Limitations of the study discussed? * |  |  |  |

* Questions in addition to Kmet
