# Supplementary material for: EEG signatures of cognitive and social development of preschool children–a systematic review
Source: PLoS One. 2021 Feb 19;16(2):e0247223. doi: 10.1371/journal.pone.0247223 (PMC7895403; doi:10.1371/journal.pone.0247223)
Supplement: S3 Table — (DOCX) [file pone.0247223.s006.docx]

**Supplementary Table S3:** Participant details of included studies

|  | **Excluded**  **(N)** | **Reasons for exclusion of EEG data** | **Recruitment method** | **Ethnicity** | **SES**^*^ |
| --- | --- | --- | --- | --- | --- |
| **Executive function: visual attention, working memory and inhibitory control** | | | | | |
| Lahat et al. (2009) | 17 | Refused to wear the EEG net – 7  Fewer than 11 correct no-go trials that that were free of eye blinks or movement artifacts- 9  Data were not recorded due to technical difficulties – 1 | Computerized database | 54% European-Canadian; 46% Chinese-Canadian | European-Canadian  85% mothers; 65% fathers had post-secondary education Chinese-Canadian  All parents had post-secondary education |
| Chevalier et al. (2014) | 10 | ERP averages included fewer than ten trials per condition - 10 | Not specified | 66.7% Caucasian, 33.3% other ethnic/racial backgrounds. | Median household income was $60,250 (M = $63,237; SD = $40, 327) and $51,209.  Maternal education: mean 16.1 years (SD = 2.8) Paternal education: mean 14.7 years (SD = 2.7) |
| Rahman et al. (2017) | 11 | poor task performance – 6  excessive EEG movement or eye-blink artifact – 5 | Local businesses that served families with young children (e.g., preschools, health offices, pediatricians) and by word of mouth. | Slow condition: 73.3% European American, 6.7% African American, and 20% mixed ethnicity children.  Fast condition: 75% European American, 6.25% African American, 6.25% Asian American, and 12.5% mixed ethnicity children. | Parent-reported health insurance status was used as a proxy for SES. 71% were middle or upper-middle SES, with private health insurance. 29% were low SES and eligible for public health insurance. |
| Hoyniak et al. (2018) | 30 month-olds – 81  36 month-olds – 49  42 month-olds - 31 | (reason – n for 30, 36, 42 month-olds)  Refused to wear cap – 16, 9, 5  Refused to do task – 19, 10, 5  Too many bad channels – 3, 4, 1  Not enough usable trials – 40, 23, 14  Other technical problem – 3, 3, 6  Total missing – 81, 49, 31 | From a mid-sized, Midwestern city, as part of a larger, ongoing multi-site longitudinal study | 90% Caucasian, 3% Latino, 2% Black, 3% Other, and 2% Unknown, Not Reported, or Missing | Primary caregiver education level: 87% college degree, 10% some college, 2% high school diploma or less, 1% not reported.  SES: calculated using the Hollingshead Four Factor Index. SES estimates ranged from 13 to 66 (M = 48.51, SD = 13.51), suggesting that the sample was predominantly middle class. |
| Brooker (2018) | None – all missing data imputed | (reason – n for 3.5-year olds, n for 4.5-year olds)  Refused to complete the task – 4, 2  Refused to be capped – 6  Joined the study at a later age - 13  Did not provide usable EEG data – 5, 7  Data unusable due to technical error – 3, 3  Did not provide enough usable incorrect trials for averaging – 6, 18  Did not provide enough usable correct trials for averaging – 7  Did not provide enough correct or incorrect trials of usable data – 17, 6  Had not yet turned 4.5 at the time of this report – 0, 1  Total missing – 61, 37 | Mailings based on local birth records, fliers, media advertisements, in person, and via word-of-mouth. | 96.2% mothers & 94.9% fathers White, 1.9% mothers & 1.3% fathers Asian, 1.91% mothers & 3.8% fathers American Indian or Alaska Native, 1.9% mothers & 5.6% fathers Hispanic or Latino | Four Factor Index of Social Status used. SES scores suggested that the average parents were skilled craftspeople, clerical, or sales workers (M = 32.10; SD = 17.46). Maternal and paternal reports were composited to assess income level. 3.03% less than $15,000, 4.04% $15,001 – $20,000, 7.07% $20,001 – $30,000, 7.07% $30,001 – $40,000, 8.08% $40,001 – $50,000, 16.16% $50,001 – $60,000, 7.07% $60,001 – $70,000, 11.11% $70,001 – $80,000, 9.09% $80,001 – $90,000, 27.27% $90,000 or greater. |
| St John (2019) | 56 | Did not understand task - 3  Declined to complete task - 6  ERPs not usable for go, no-go trials – 29  EEG technical difficulties – 5  Braided hair (prevented electrodes from contact with scalp – 4  Declined to wear EEG cap - 9 | Departmental database of families interested in research; from publicly available state birth records; from online advertising; and through face-to-face- recruitment events. | 46.40% White, 7.20% Black,  7.20% Hispanic/Latino,  14.50%  Asian, 24.60% Multiracial | Income-to-needs ratio: 4.88 (3.72) Parent Education Average: 4.03(1.03)  Maternal Education: Middle school or some high school - 4.30%  High school graduate or GED - 4.30%  Some college - 11.60%  4-year college degree - 29.00% Graduate degree - 50.70% |
| Rueda et al. (2004) | 8 | Did not pass data cleaning thresholds - 8 | Not specified | Not specified | Not specified |
| Begnoche et al. (2016) | 25 | Withdrew from the parent project – 1  Failed to respond to the invitation to participate – 7  Moved away from the area – 3  Declined to participate – 13  Did not show for their laboratory visit and did not reschedule – 1  Left-handed – 6  Did not provide usable baseline data – 4 either due to technical error (n = 1), task refusal (n = 2) or excessive artifact (n = 1)  ΔERN could not be scored - 5  Did not complete the follow-up measure – 5 | Not specified | 87.5% Non-Hispanic Caucasian, 5.0% African-American, 5.0% Asian American, 2.5% Hispanic | Annual incomes ranging from less than $15,000 to over $60,000. 47.5% reported annual incomes of more than $60,000. |
| Ruberry et al. (2016) | Flanker - 57, frogs/fish - 46 | Insufficient number of artifact-free ERP trials (flanker – 44, frogs/fish – 38)  Child fatigue or noncompliance (flanker – 10, frogs/fish – 3)  Insufficient performance on the task (fewer than six correct trials per condition) (flanker – 3, frogs/fish - 4)  Technical issues during EEG collection (frogs/fish - 1) | From a region in the Pacific Northwest via the university hospital birth records, schools, community centers, churches, and daycares. | Not specified | The following measures used (Liket scales): 1. three income categories: at or near poverty, lower income, or middle to upper income (collapsing the two highest income levels). 2. financial security or strain |
| Morasch & Bell (2011) | 23 | Rejected EEG - 20  Missing behavioural data – 3 | Not specified | 91.4% Caucasian, 4.9% African-American, 1.2% Asian, and 2.5% Hispanic | Both parents: completed a high school education, 76% had a college degree. |
| Espinet et al. (2012) | 42 | Failed to perform better than chance on the pre-switch phase of the DCCS and/or did not unambiguously pass or fail the post-switch phase – 24  Inattentive and moved or vocalized excessively – 10  ERP data did not contain 15 or more usable post-switch trials – 8 | Database of parents who had expressed interest in participating in studies and through advertisements posted in the community. | 82% Caucasian | Middle to upper-middle class backgrounds |
| Blankenship et al. (2018) | 1 | Did not have EF data due to parental interference - 1 | Participant databases and flyers distributed in areas populated by young families, including parks, recreation centres, and day-care facilities | 7.3% identified as Hispanic, 78% non-Hispanic, 14.6% did not report ethnicity. 4.9% Asian, 2.4% Black or African American, 9.8% Other/Multiracial, 82.9% Caucasian. | 75.6% of mothers, 73.2% of fathers had completed a college degree or higher (6 sets of parents did not report education). |
| Lo et al. (2013) | 4 | Did not pass inclusion criteria of 85% correct trials – 4 | Three kindergartens | Not specified | Child education: Kindergarten |
| Elke & Wiebe (2017) | 21 | Excessive artifact in the EEG data – 6  Refusal of the EEG net – 5  Technical difficulties – 4  Refusal to perform the task – 3  Early task discontinuation – 3 | Fliers and online advertising. | 51% European-Canadian. | Middle- to upper-middle class backgrounds, median annual family income of $100,000.  Both parents: Median 16 years education |
| Wolfe & Bell (2004) | 5 | Refusing to wear the EEG cap - 2  Refusing the application of EEG gels - 1  Refusing to complete the WMIC tasks - 1  EEG equipment failure - 1 | Birth announcements placed in the local newspaper | 100% Caucasian | Both parents: 68.75% had a college education Maternal education duration: mean 15.60 years Paternal education duration: mean 16.05 years |
| Bell & Wolfe (2007) | 7 | Refusing to wear the EEG cap - 2  Refusing the application of EEG gels - 1  Refusing to complete the WMIC tasks - 1  EEG equipment failure – 1  No EEG data at infancy - 2 | Birth announcements placed in the local newspaper. | 100% Caucasian | Both parents: at least a high school diploma Maternal education: 79% had college degrees Paternal education: 82% had college degrees |
| Wolfe & Bell (2007) | 22 | Refused to participate in the WMIC tasks - 7 Refused EEG - 15 | From the community | 85% European Caucasian | Both parents: mean education level over 16 years Child education: 90% had attended preschool |
| Wolfe & Bell (2007) | 30 | Loss to follow up - 25 | Birth announcements placed in local newspaper. | 100% Caucasian | Both parents: at least a high school diploma  Maternal education: 79% had college degrees  Paternal education: 82% had college degrees |
| Watson & Bell (2013) | 4 | Diagnosed with developmental delay - 1 Refused to participate - 3 | From a rural university community using a commercial list of new parent names and addresses | 4.4% Hispanic, 95.6% Non-Hispanic; 91.2% Caucasian, 1.5% African American, 7.3% Multi-Racial | Maternal education: All had at least a high school diploma. 69% had college degrees Paternal education: 96% had at least a high school diploma. 58% had college degrees |
| Wolfe & Bell (2014) | 24 | Contributed no shyness data (due to parents not returning the CBQ) - 3 Refused cap application – 16  Excessive artifact - 1 Contributed baseline EEG data but not task - 7 | Participants from two other studies, recruitment method not specified | 89% European Caucasian | Both parents: at least a high school diploma, 66.5% had a college degree |
| Cuevas et al. (2016) | 46 | Behavioural task refusal/non-compliance – 17  Failure to pass practice trials – 9  Parental interference – 3  “non-Stroop” version not in protocol- 3  Experimenter error – 1  Did not accept EEG electrodes - 7  Experiment failure - 3  Insufficient artifact free data - 3 | Commercial mailing lists, newspaper birth announcements, and word of mouth. | 6.3% Hispanic, 93.7% Non-Hispanic; 79.2% Caucasian, 14.6% African American, 2.8% Other Race; 2% Multi-Racial, 1.4% Not Reported | Both parents: those reported educational information (139 mothers, 136 fathers), 97.8% completed a high school education (7.2% and 8.8% technical degree; 46.8% and 33.1% bachelor’s degree; 22.3% and 22.8% graduate degree). |
| Swingler et al. (2011) | 8 for EEG. 19 for power; 20 for coherence | EEG data not collected – 8  Unusable EEG data - (power 19) (coherence 20) | From the community through advertisements in local and university newspapers and through flyers posted at local childcare centers. | EEG: 79% Caucasian power: 81% Caucasian coherence: 80% Caucasian | Income: Mean in dollars (SD) 71,057 (28,503), predominantly middle class backgrounds |
| **Selective auditory attention** | | | | | |
| Bartgis et al. (2003) | 10 | Too few artifact free trials- 7 Equipment failure- 1 Lack of cooperation - 2 | Birth announcements published in a local newspaper and through referrals from the parents of other participants. | Not specified | Not specified |
| Sanders et al. (2006) | 16 | Artifacts - 7 Failing to attend - 4 Matching group sample sizes - 5 | Not specified | Not specified | Lower middle to upper middle class with an average of middle class for all groups on the Hollingshead Index of Social Position. |
| Pesonen et al. (2010) | 13 | Behavioral assessments and home observations were not completed- 8 Unsuccessful ERP recordings - 5 | Not specified | Not specified | Upper middle-class background  Maternal education: majority had a university degree Child education: enrolled in a music play school (specialized in pop and jazz) |
| Sanders and Zobel (2012) | 2 | insufficient number of artifact-free trials (> 45) - 2 | Not specified | Not specified | Not specified |
| Strait et al. (2014) | Not specified | n/a | Not specified | Not specified | Not specified |
| Karns et al. (2015) | 0 | n/a | Developmental Database of the University of Oregon | Not specified | Maternal education: mean 6.1 (6=masters level; 7=doctorate) |
| Isbell et al. (2016) | 34 | Low ERP data quality (excessive EEG artifacts and/or less than 75 trials per condition) – 23  Less than 50% accuracy on the comprehension questions presented during the ERP task - 11 | From 12 Head Start (HS) preschool sites in Oregon, a program for families living at or below the poverty line. | 58% White/Caucasian, 1% Black/African American, 4% American Indian, 16% more than one ethnicity, 21% unreported. | At least 90% of the enrolled children from low-income families |
| Wray et al. (2017) | 11 | Too few trials per condition – 1 in LSES group Answered fewer than half of the ERP paradigm comprehension questions – 10 in LSES group | LSES group from Head Start preschool sites in Oregon as part of an ongoing longitudinal study, HSES group from the Developmental Database at the University of Oregon | LSES group: 72.7% Caucasian, 6.1% American Indian/Alaskan Native, 3% Asian, and 15.2% bi/multiracial, 3% chose not to respond.  HSES group: 78.6% Caucasian, 7.1% not Hispanic/Latino (but without identifying race), 14.3% chose not to respond | Hollingshead Four Factor Index used (range = 8 – 66) HSES group: 52.36 (1.57); LSES group: 30.16 (2.06)  Maternal education: High SES: 6.07 (.16); Low SES: 4.94 (.15) Paternal education: High SES: 6.43 (.17); Low SES: 4.39 (.17) (2 = 9th grade completed; 3 = 10–11th grade completed/partial high school; 4 = high school graduate; 5 = partial college; 6 = college graduate; 7 = graduate degree) |
| Giuliano et al. (2018) | 19 | Unusable or declined measurement of electrocardiogram (ECG) – 17  Declined to report socioeconomic information - 2 | Currently enrolled in Head Start | 71% white or Caucasian, 7% Hawaiiaan or Pacific Islander, 1% American Indian or Alaskan Native, 1% Black or African American, 20% mixed race. | Socioeconomic Risk Subgroups calculated using: household income, people in household, marital status, maternal education. |
| **Learning and memory** | | | | | |
| Marshall et al. (2002) | 16 | Equipment failure – 2  Excessive artifact – 6  General fussiness and failure to engage in the task – 4  Low performance – 4 | Local preschools in the suburban Washington, DC area. | 50% Caucasian, 20% African American, 25% biracial, 5% Asian. | Middle-class background |
| Riggins et al. (2009) | 10 | Electrophysiological data were excluded as a result of:  Refusal to wear the cap – 5  Excessive movement artifact resulting in an insufficient number of trials – 2  Missed appointment – 1  Equipment failure – 2 | Participants’ mothers were recruited before giving birth for participation in an ongoing longitudinal research project examining cognitive development | not specified - details of cohort (Nelson 2007: Nelson CA, Thomas KM, de Haan M. Neural bases of cognitive development. In: Mussen P, editor. Handbook of child psychology. New York: John Wiley & Sons; 2007. pp. 3–57) | not specified - details on sample (Nelson 2007) |
| Riggins & Rollins (2015) | Not specified | participants with fewer than 10 trials were excluded | Not specified | 63% White/Caucasian, 17% Black/African American, 5% Asian, 9% multiracial, 5% did not disclose | Not specified |
| Canada et al. (2019) | 33 | Did not provide usable behavioral data – 7  Less than 10 ERP trials in the conditions of interest, resulted from a combination of movement artifact and low trial numbers – 26 | Database of interested families from a major metropolitan area and the distribution of recruitment flyers | 75% Caucasian  Others not specified | 75% middle- to high-income households (median income greater than $105,000 per year)  Others not specified |
| Meyer et al. (2014) | 7 | Unwilling to wear the EEG cap – 1  Did not provide a sufficient amount of artifact-free EEG trials that were also behaviorally valid trials – 6 | Database of interested families | Not specified | Not specified |
| **Face processing** | | | | | |
| Taylor et al. (2001) | 20 | Insufficient number of artefact-free trials or in a few cases due to technical problems with data collection. | Not specified | Not specified | Not specified |
| Peykarjou et al. (2013) | 41 | Excessive eye and/or body movements that resulted in recording artifact - 23  Too few trials were recorded for inclusion – 3  Equipment failure or experimenter error – 7  Refusal to wear the sensor net - 8 | Not specified | Not specified | Not specified |
| Lochy et al. (2019) | 0 | n/a | Not specified | Not specified | Child: Third year of Kindergarten |
| Lochy et al. (2020) | 2 | Extremely noisy data on all electrodes  or on posterior electrodes – 2 | Two schools (Brabant-Wallon region, Belgium) | 82.7% European Caucasian, 9.6% Middle Oriental, 5.8% mixed Caucasian-African, 1.9% African | High socioeconomic status |
| Meaux et al. (2014) | 4 | Excessive ocular and/or muscular artifacts and unsatisfactory attempts during the electroencephalographic (EEG) experiment – 2  Gaze tracking signal was not sufficient during the eye-tracking experiment - 2 | Not specified | Not specified | Not specified |
| Melinder et al. (2010) | 7 | Did not pass artifact rejection - 7 | Not specified | 100% Scandinavian Caucasian | Not specified |
| Carver et al. (2003) | 23 | Did not provide enough artifact-free data for analysis – 23  Did not cooperate with the testing procedure – 9  Equipment failure during testing – 4  Below-average cognitive functioning – 1 | Not specified | 95.6% European American, 2.2% African American, 2.2% Asian American. | Middle-class background |
| **Emotional stimuli processing – faces** | | | | | |
| Batty and Taylor (2006) | total 13 4-5 yr olds 6 | Did not finish the entire study - 2  Too much ocular and/or muscular artefact and an insufficient number of good trials - 11 | Local schools and colleagues | Not specified | Not specified |
| Vlamings et al. (2010) | 7 | Ocular or muscular artefacts or an insufficient number of trials in which they looked at the screen - 7 | Elementary school in Kerkrade (Netherlands). | Not specified | Child: elementary school |
| Jiang et al. (2017) | 10 | <15 segments of good EEG data in one experimental condition – 10 | Not specified | Not specified | Not specified |
| **Emotional stimuli processing – non-faces** | | | | | |
| Theall-Honey and Schmidt (2006) | 4 | Equipment failure or excessive artifact in the data – 4 (2 shy, 2 non shy) | Child database of births at McMaster University Medical Centre and St. Joseph’s Hospital in Hamilton, Ontario. Then 40 children who fell in the top and bottom 15% on the CCTI shyness scale after screening were recruited | Primarily Caucasian | Middle-class background and from the Hamilton area. |
| Cheng et al. (2014) | 22 | Insufficient artifact-free trials (<20) – 22  Fell asleep - 3  Dropped out before behavioral data were collected - 4  Failed to concentrate on experimental procedures - 15 | Not specified | Not specified | Not specified |
| Hua et al. (2014) | 0 | n/a | Not specified | 100% Chinese | Not specified |
| Hua et al. (2015) | 5 | Failure to complete the EEG portion of the study, lack of ability to focus attention on the screen, or excessive blink and movement artifact in the EEG recordings - 5 | Not specified | 100% Chinese Han | Middle-class background |
| Mai et al. (2011) | 5 | Equipment problems - 1  could not sit still - 1 Technical errors or excessive data artifacts - 3 | Not specified | Not specified | Not specified |

* Abbreviation **-** SES: Socioeconomic status including income and parental and child education
