# Supplementary material for: EEG signatures of cognitive and social development of preschool children–a systematic review
Source: PLoS One. 2021 Feb 19;16(2):e0247223. doi: 10.1371/journal.pone.0247223 (PMC7895403; doi:10.1371/journal.pone.0247223)
Supplement: S4 Table — (DOCX) [file pone.0247223.s007.docx]

**Supplementary Table S4**: Data acquisition setup of included studies

|  | **Brand** | **Number of channels** | **Sampling rate** | **Max impedance** | **Data acquisition software** |
| --- | --- | --- | --- | --- | --- |
| **Executive function: visual attention, working memory and inhibitory control** | | | | | |
| Lahat et al. (2009) | Electrical Geodesics, Inc. (Hydrocel Geodesic Sensor Net) | 128 | 1000 Hz | Below 40 Kohms | Netstation 4.1.2 (Electrical Geodesics, Inc.) |
| Chevalier et al. (2014) | Electrical Geodesics, Inc. (Hydrocel Electrical Geodesic Sensor Net) | 128 | 250 Hz | Below 50 Kohms | Net Station 4.3.1 (Electrical Geodesics, Inc.) |
| Rahman et al. (2017) | Electrical Geodesics, Inc. (Hydrocel Geodesic Sensor Net) | 128 | 250 Hz | Below 50 Kohms | Not specified |
| Hoyniak et al. (2018) | Electrical Geodesics, Inc. (Hydrocel Geodesic Sensor Net) | 128 | 250 Hz | Below 50 Kohms | Netstation Acquisition Software |
| Brooker (2018) | BioSemi (Active Two system) | 64 | 2048 Hz | Not specified | Active Two system (Biosemi). |
| St John (2019) | Electrical Geodesics, Inc. (Hydrocel Geodesic Sensor Net) | 128 | 500 Hz | Not specified | Netstation Acquisition Software |
| Rueda et al. (2004) | Electrical Geodesics, Inc. (Geodesic Sensor Net) | 128 | 250 Hz | Below 80 Kohms | Net Station 2.0 (Electrical Geodesics, Inc.) |
| Begnoche et al. (2016) | Electrical Geodesics, Inc. (Hydrocel Geodesic Sensor Net) | 128 | 500 Hz | Below 80 Kohms | NetStation (v 4.3.1) (Electrical Geodesics, Inc.) |
| Ruberry et al. (2016) | Biosemi (Active Two system) | 32 | 256 Hz | Below 25 µV | Active Two system (Biosemi). |
| Morasch & Bell (2011) | Electro-Cap, Inc. | 16 | 512 Hz | Below 10 Kohms | Not specified |
| Espinet et al. (2012) | Electrical Geodesics, Inc. (Geodesic Sensor Net) | 128 | 250 Hz | Below 80 Kohms | NetStation 4.1.2 (Electrical Geodesics, Inc.) |
| Blankenship et al. (2018) | Electro-Cap, Inc. | 26 | Not specified | Below 20 Kohms | Snapshot-Snapstream (HEM Data Corp., Southfield, MI) |
| Lo et al. (2013) | NeuroScan Synamp2 | 32 | 500 Hz | Below 10 Kohms | Not specified |
| Elke & Wiebe (2017) | Electrical Geodesics, Inc. (Hydrocel Geodesic Sensor Net) | 128 | 250 Hz | Below 50 Kohms | NetStation 4.4.2 (Electrical Geodesics, Inc.) |
| Wolfe & Bell (2004) | Electro-Cap, Inc. | 16 | 512 Hz | Below 5 Kohms | Snapshot-Snapstream (HEM Data Corp.) |
| Bell & Wolfe (2007) | Electro-Cap, Inc. | 16 | 512 Hz | Below 5 Kohms | Snapshot-Snapstream (HEM Data Corp.) |
| Wolfe & Bell (2007) | Electro-Cap, Inc. | 16 | 512 Hz | Below 10 Kohms | SnapMaster |
| Wolfe & Bell (2007) | Electro-Cap, Inc. | 16 | 512 Hz | Below 5 Kohms | Snapshot-Snapstream (HEM Data Corp.) |
| Watson & Bell (2013) | Electro-Cap, Inc. | 16 | 512 Hz | Below 20 Kohms | Snapshot-Snapstream (HEM Data Corp.) |
| Wolfe & Bell (2014) | Electro-Cap, Inc. | 16 | Not specified | Below 10 Kohms | Not specified |
| Cuevas et al. (2016) | Electro-Cap, Inc. (E1-series cap) | 16 | 512 Hz | Below 20 Kohms | Snapshot-Snapstream (HEM Data Corp) |
| Swingler et al. (2011) | Electro-Cap, Inc. | 16 | 512 Hz | Below 20 Kohms | Snapshot-Snapstream (HEM Data Corp.; Southfield, MI). |
| **Selective auditory attention** | | | | | |
| Bartgis et al. (2003) | Not specified | 8 | 200Hz | Below 10 Kohms | Not specified |
| Sanders et al. (2006) | Electro-Cap, Inc. | 29 | 250 Hz | Eye electrode: below 10 Kohms  Mastoid and scalp electrodes: below 5 Kohms | Not specified |
| Pesonen et al. (2010) | Neuroscan | 5 | 500 Hz | Not specified | Not specified |
| Sanders and Zobel (2012) | Electrical Geodesics, Inc. | 128 | 250 Hz | Not specified | Electrical Geodesics, Inc. |
| Strait et al. (2014) | Electro-Cap, Inc. | 14 | 500 Hz | Below 20 Kohms with less than 5 Kohms difference across channels. | NeuroScan Acquire 4.3 (Compumedics) |
| Karns et al. (2015) | Biosemi (Active-Two system) | 32 | 512 Hz | Not specified | Not specified |
| Isbell et al. (2016) | Biosemi (Active-Two system) | 32 | 1024 Hz | system does not require impedance measurements | Not specified |
| Wray et al. (2017) | Biosemi (Active-Two system) | 32 | 512 Hz and downsampled offline to 256 Hz. | Not specified | Not specified |
| Giuliano et al. (2018) | Biosemi (Active-Two system) | 32 | 512 Hz | Not specified | BioSemi Active 2 |
| **Learning and memory** | | | | | |
| Marshall et al. (2002) | Not specified | 10 | 512 Hz | Below 5 Kohms | Not specified |
| Riggins et al. (2009) | not specified | 32 | 100 Hz | Below 10 Kohms | Not specified |
| Riggins & Rollins (2015) | Biosemi (Active-Two system) | 64 | 512 Hz | Not specified | BioSemi Active 2 |
| Canada et al. (2019) | Biosemi (Active-Two system) | 64 | 512 Hz | Not specified | BioSemi Active 2 |
| Meyer et al. (2014) | Brain Amp (ActiCAP) | 32 | 500 Hz | Below 25 Kohms | Brain Vision Recorder (Brain ProductsGmbH). |
| **Face processing** | | | | | |
| Taylor et al. (2001) | ECI electrode cap, NeuroScan system | 29 | Not specified | Not specified | NeuroScan |
| Peykarjou et al. (2013) | Electrical Geodesics, Inc. (Geodesic Sensor Net) | 124 | 250 Hz. | At or below 100 Kohms | NetStation 4.3 (Electrical Geodesics, Inc.) |
| Lochy et al. (2019) | Biosemi (Active-Two system) | 32 | 1024 Hz | Not specified | Not specified |
| Lochy et al. (2020) | Biosemi (Active-Two system) | 32 | 1024 Hz | Not specified | Not specified |
| Meaux et al. (2014) | Not specified | 18 | 500 Hz | Below 5 Kohms | Compumedics NeuroScan EEG system (Synamps amplifier, Scan 4.3 and Stim2 software) |
| Melinder et al. (2010) | Electrical Geodesics, Inc. (Hydrogel Geodesic Sensor Net) | 128 | 250 Hz | At or below 100 Kohms | NetStation (Electrical Geodesics, Inc.). |
| Carver et al. (2003) | Electrical Geodesics, Inc. (Geodesic Sensor Net) | 64 | 250 Hz | Below 40 Kohms | Not specified |
| **Emotional processing – faces** | | | | | |
| Batty and Taylor (2006) | Easycap electrode cap (FMS Falk Minow) | 30 | 500 Hz | Below 5 Kohms | Not specified |
| Vlamings et al. (2010) | Compumedics (QuickCap) | 37 | 500 Hz | Below 20 Kohms | QuickCap (Compumedics) |
| Jiang et al. (2017) | Electrical Geodesics, Inc. (Geodesic Sensor Net) | 128 | 250 Hz | Below 50 Kohms | Not specified |
| **Emotional stimuli processing – non-faces** | | | | | |
| Theall-Honey and Schmidt (2006) | Electro-Cap, Inc. | 6 | 512 Hz | Below 10 Kohms | Not specified |
| Cheng et al. (2014) | Neuroscan Inc. (NuAmps) | 9 | 512 Hz | Below 5 Kohms | Not specified |
| Hua et al. (2014) | NeuroScan Inc. | 40 | 1000 Hz | Not specified | NeuroScan, Inc., USA |
| Hua et al. (2015) | NeuroScan Inc. | 40 | 1000 Hz | Not specified | Not specified |
| Mai et al. (2011) | Electrical Geodesics, Inc. (Geodesic Sensor Net) | 128 | 250 Hz | Below 50 Kohms | Not specified |
