# Supplementary material for: EEG signatures of cognitive and social development of preschool children–a systematic review
Source: PLoS One. 2021 Feb 19;16(2):e0247223. doi: 10.1371/journal.pone.0247223 (PMC7895403; doi:10.1371/journal.pone.0247223)
Supplement: S5 Table — (DOCX) [file pone.0247223.s008.docx]

**Supplementary Table S5**: Data preprocessing details of included studies

|  | **Data analysis software** | **Data preprocessing** | |
| --- | --- | --- | --- |
| **Executive function: visual attention, working memory and inhibitory control** | | | |
| Lahat et al. (2009) | Not specified | Artifacts: Eye blinks, eye movements, movement artifacts removed using >200 μV, and fast transits >100 μV  Filters: Band passed 1–30 Hz  Reference: Cz during recording, offline re-referenced to average  Segment length: -200 to 1000 ms, baseline corrected  Window: N2 was the largest negative deflection after the P1 with a peak latency of 250–500 ms post-stimulus.  Electrodes: Data averaged across right and left-hemisphere fronto-central cluster encompassing F4 and F3  Trials containing >20% artifacts eliminated  Source model: minimum norm method with LAURA | |
| Chevalier et al. (2014) | Not specified | Artifacts: Eye blinks removed using ICA in EP Toolkit 2.23 to remove and reconstitute components that correlated ≥.9 with the scalp topography of the blink template. Bad channels: amplitude within a trial varied by >100 μV or maximum difference from the most similar neighboring electrode >30 μV. Channels rejected if >20% trials were bad, trials rejected if ≥ 10% channels were bad. Bad channels replaced using spline interpolation based on neighboring channels.  Filters: 30 Hz low-pass filter  Reference: Cz during recording, offline re-referenced to average  Segments: 850 ms (-100 to 750ms), baseline corrected  Electrodes: a spatial PCA identified the peak electrodes contributing to the LFN and peak amplitudes and latencies were extracted by averaging across these electrodes  Trials: response time >200 ms discarded | |
| Rahman et al. (2017) | Net Station v4.3.1) | Artifacts: Bad electrodes removed using signal fluctuations >200 μV, replaced after spline interpolation. Eye movements removed using EOG correction using Gratton’s algorithm and visual inspection  Filters: Band passed 0.1–100 Hz. Band passed 0.3–30 Hz offline.  Reference: Cz during recording, offline re-referenced to average using polar average reference effect correction  Segment length: 1300 ms (-100 to 1200 ms), baseline corrected Segments with >12 bad electrodes rejected. Trials: Incorrect trials and trials with RT <200 ms excluded, minimum of 10 artifact-free trials in each trial type | |
| Hoyniak et al. (2018) | Netstation Acquisition Software | Artifacts: Manually and then automatically examined for artifacts (removing channels that contained a voltage shift greater than 150 μV during a given segment of length 80 ms, and removing epochs that contained 20 or more bad channels). Removed channels were then interpolated based on the waveforms of surrounding electrodes.  Filers: Band-pass filtered from 0.3 to 30 Hz  Reference: Re-referenced to the average of all scalp electrodes  Segment length: 1200 ms (-200 – 1000 ms), baseline corrected  Electrodes: Frontocentral electrode group identified by temporospatial PCA  Window: 350 – 450 ms post stimulus identified by temporospatial PCA  The child had to have at least 8 correct, artifact-free trials in each condition | |
| Brooker (2018) | Not specified | Artifacts: EOG correction using Gratton’s algorithm. Artifacts defined as a voltage step of more than 75 μV between data points, a difference of 150 μV per within 200ms, amplitudes below 0.5 μV within a 50ms period, and activity that exceeded +100μV or −100 μV and visual inspection.  Filers: High-pass filtered at 0.1 Hz, low-pass filtered at 30 Hz  Reference: CMS and DRL electrodes, offline re-referenced to the average of the right and left mastoids  Segment length: 800ms (−200 to 600 ms), baseline corrected  Electrodes: FCz  Window: peak negative amplitudes between 0 and 100 ms  Trials: At least 6 trials of usable data | |
| St John (2019) | Netstation | Artifacts: Automatic rejection of artifacts, then manual rejection of segments with with eye blinks (>140μV differential average) or eye movements (>100μV differential average). Bad channels were replaced via interpolation. Segments for each child were averaged within each trial type (go and no-go)  Filers: Band-pass filtered from 0.3 to 30 Hz  Reference: Re-referenced to the average reference  Window: 400-700ms post-stimulus  The child had to have at least 10 useable trials for each trial type to be included in the analysis | |
| Rueda et al. (2004) | Net Station 3.0. | Artifacts: Eye blinks and movements removed using Artifact Detection NS tool, threshold 100 μV, Channel average amplitude ≥ 200 μV or difference average amplitude ≥ 100 μV discarded, Channels with artifacts in > 50% segments discarded  Filters: Band passed 1-12 Hz  Reference: Cz during recording, offline re-referenced to average  Segment length: 1.7 sec (-200 to 1500 ms), baseline corrected  Window (adults and children): N1 - 50–150 ms and 100–200 ms, N2 - 200–400 ms and 300–550, P3 - 250–650 ms and 400–1100 ms; LPC - 550–1300 in children.  Segments: > 25 bad channels rejected Participants: > 12 clean segments per flanker condition among the correctly responded trials included | |
| Begnoche et al. (2016) | Brain Vision Analyzer (Brain Products) | Artifacts: Eye movement or eye blink removed using ICA in EEGLab Version 8.0.3b, step>75μV or difference > 150 μV or absolute voltage >200 μV within a segment, or amplitudes <0.5 μV within a 50 ms period. Also, visual inspection.  Filters: Band passed 0.1-100 Hz, Lowpass filtered at 30 Hz  Reference: Cz during recording, offline re-referenced to average of two mastoid channels.  Error related negativity Segment length: 1600 ms (-600 to 1000 ms), baseline corrected  Difference wave: error trial average - correct trial average. Peak ΔERN mean amplitude (+/-20 ms) around the most negative peak in -100 to 100 ms.  Electrodes: Fz, Cz, and Pz  Alpha asymmetry Transformation: FFT using a Hamming window Segment: 1.024 s width and 50% overlap Segment  Electrodes: frontal (Fp1, Fp2), and parietal (P3, P4) Baseline episodes collapsed  Trials: response times <200 ms removed | |
| Ruberry et al. (2016) | EEGLab and ERPLab | Artifacts: Eye blinks or movement removed using visual inspection  Filters: Band passed 0.01-30 Hz.  Reference: Correct trial data referenced to Cz  Segment length: –200 to 1500 ms, baseline corrected Window: N2 - flanker task 250-450 ms post stimulus, frogs/fish task 250-400 ms; both tasks P3 400-700 ms post stimulus  Electrodes: N2 at frontal sites (F3, F4, Fz), P3 at parietal sites (P3, P4, Pz)  Epochs: voltage difference between sensors >100 mV in six or more sensors excluded | |
| Morasch & Bell (2011) | James Long Company software | Artifacts: Eye movements removed using peak to peak ≥ 100 uV. Movement artifacts removed using peak to peak ≥ 200 uV. Power imputed for bad electrodes using average power value of the two most proximal ipsalateral scalp sites to the missing electrode summed with the averaged deviation from these two neighboring scalp sites.  Filters: not specified  Reference: Cz during recording  Transformation: DFT using a Hanning window  Segment: 1-second width and 50% overlap | |
| Espinet et al. (2012) | N2 topography - NetStation GeoSource 2.0 | Artifacts: Bad channels replaced through interpolation of neighboring electrodes if the fast average amplitude >200 V; differential average amplitude >100 V; and/or channel variance=0. Eye movement corrected using ocular artifact removal tool in Net Station (EOG sensitivity =1.35 V).  Filters: Band passed 1–30 Hz  Reference: Cz during recording, offline re-referenced to average  Segment length: -400 to 700 ms, baseline adjusted using 200ms prior to stimulus Trials: visual inspection for good/bad trials Segments: excluded if >25 bad channels and/or eye blinks or eye movements. | |
| Blankenship et al. (2018) | James Long Company software | Artifacts: Average reference EEG data were artifact scored for eye movements using a peak-to-peak criterion of 100μV or greater. Gross motor movements over 200μV peak to peak were also scored, and all artifact scored epochs were excluded  Reference: Cz during recording  Transformation: DFT using a Hanning window  Electrodes: F3/F4 electrode sites | |
| Lo et al. (2013) | SPM8 for MEG/EEG | Artifacts: Eye movements: Vertical and horizontal EOG used to derive eye blink peaks using regression and correlation and data corrected. Epochs with artifacts >±100 μV rejected. Time series analysis - trial artifacts >±150 μV excluded.  Filters: Band passed 0.05–70 Hz. ERP: A digital low-pass filter of 30 Hz  Reference: Referenced to average of left and right mastoids, ground electrode placed between FPz and Fz  Segment length: -100 to 800 ms, baseline corrected  Conditions: > 12valid trials  Continuous EEG data (-1000 to 1000ms)  Transformation: Morlet wavelet transform (i.e., mf0σt = 7) from 2 to 65 Hz | |
| Elke & Wiebe (2017) | EEGlab, MATLAB | Artifacts: Bad channels and epochs removed using visual inspection, amplitude > 100 μV, great deviation from neighbors or substantial oscillatory noise. Eye, muscle, and line-noise artifacts removed using ICA, removed channels spherically interpolated.  Filters: Band passed 0.1–30 Hz  Reference: Cz during recording, offline re-referenced to average  Segment length: -200 to 3600 ms, baseline corrected  Cue processing epoch -200 to 1400 ms, stimulus processing epoch -200 to 1400 ms  First trial of each block, trials with RTs <200 ms, those occurring after an error excluded | |
| Wolfe & Bell (2004) | James Long Company | Artifacts: Eye movements removed using EOG, movement artifact removed using visual inspection  Filters: Not specified  Reference: Cz during recording, offline re-referenced to average  Transformation: DFT using a Hanning window  Segment: 1-second width and 50% overlap | |
| Bell & Wolfe (2007) | James Long Company | Artifacts: Eye movements removed using peak to peak ≥100 uV, eye blinks removed using visual inspection, movement artifact removed using peak to peak ≥ 200 uV  Filters: Band passed 1-100 Hz  Reference: Cz during recording, offline re-referenced to average  Transformation: DFT using a Hanning window  Segment: 1-second width and 50% overlap  Electrodes: Coherence computed between medial frontal and all other electrode sites within each hemisphere  Tasks: Day-Night and Yes-No tasks combined by weighting the amount of artifact-free EEG collected during each task. | |
| Wolfe & Bell (2007) | James Long Company | Artifacts: Eye blinks removed using visual inspection of Fp1 and Fp2, Gross motor and muscle movements removed using visual inspection  Filters: Band passed .1-100 Hz  Reference: Cz during recording, offline re-referenced to average  Transformation: DFT using a Hanning window  Segment: 1-second width and 50% overlap | |
| Wolfe & Bell (2007) | James Long Company | Artifacts: Eye movements removed using peak to peak≥100 uV, movements artifacts removed using peak to peak≥200 uV  Filters: Not specified  Reference: Cz during recording, offline re-referenced to average  Transformation: DFT using a Hanning window  Segment: 1-second width and 50% overlap  Tasks: Power values from the day–night task and the yes–no task averaged | |
| Watson & Bell (2013) | James Long Company | Artifacts: Eye movements removed using peak to peak≥100uV, movement artifact removed using peak to peak≥200 uV  Filters: Not specified  Reference: Cz during recording, offline re-referenced to average  Transformation: DFT using a Hanning window  Segment: 1-second width and 50% overlap  Participants: artifact-free DFT windows <10 excluded | |
| Wolfe & Bell (2014) | James Long Company | Artifacts: Eye blinks removed using peak to peak≥ 100 uV, movement artifacts removed using peak to peak ≥ 200 uV  Filters: Band passed 1-100 Hz  Reference: Cz during recording, offline re-referenced to average  Transformation: DFT using a Hanning window  Segment: 1-second width and 50% overlap | |
| Cuevas et al. (2016) | James Long Company | Artifacts: Eye movements removed using peak to peak≥100 uV, movement artifacts removed using peak to peak≥200 uV  Filters: Band passed .1-100 Hz  Reference: Cz during recording, offline re-referenced to average  Transformation: DFT using a Hanning window  Segment: 1-second width and 50% overlap  Electrodes: averaged power across hemispheres for frontal pole, medial frontal, and lateral frontal  Difference score: “Stroop-like” – “non-Stroop” | |
| Swingler et al. (2011) | James Long Company | Artifacts: Eye blinks and movement artifacts using visual examination  Filters: Band passed .1-100 Hz  Reference: Cz during recording, offline re-referenced to average  Transformation: DFT using a Hanning window  Segment: 1-second width and 50% overlap  Difference scores: task power-baseline power for each electrode site  Electrodes: Intrahemispheric coherence computed within the frontal and prefrontal electrode sites and between the frontal and occipital electrode sites within each hemisphere. | |
| **Selective auditory attention** | | | |
| Bartgis et al. (2003) | Not specified | Artifacts: Any channel >100 μV  Filters: Band passed 0.1–100 Hz. Digitally filtered at 50 Hz.  Reference: Referenced to linked earlobes, ground located on forehead.  Segment length: -50 to 600ms, baseline corrected  Electrodes: Cz, Fz, and Pz  Window: mean amplitude P3 - 300- to 600ms, Nd - 0 to 600ms  Difference waves: Nd waves ERP generated to standards in attended minus ignored ear  Participants <60 artifact-free standard and 20 target trials excluded. Reaction times <200 ms | |
| Sanders et al. (2006) | Not specified | Artifacts: Eye movements or muscle activities removed using Visual inspection  Filters: 60 Hz digital filter  Reference: Right mastoid; re-referenced to averaged mastoids offline  Segment length: -100 to 500ms, baseline corrected Participants: >40 artifact-free trials in each condition  Windows: mean amplitude for 100–200 ms, 200–300 ms, and 300–450 ms epochs.  Electrodes: across 24 electrode sites | |
| Pesonen et al. (2010) | Not specified | Artifacts: Eye movements removed using EEG or EOG signals >125 mV discarded  Filters: Band passed 0.1–95 Hz. Band passed 0.5–30 Hz after recording.  Reference: Fpz during recording, re-referenced to average of mastoids offline  Window: 210–290 ms after repeated and non-repeated novel sounds onset, two 80-ms time windows, 115–195 and 235–315 ms after standard stimulus onset  Electrodes: mean amplitude of F3, F4, C3, and C4 standard stimuli | |
| Sanders and Zobel (2012) | Not specified | Artifacts: Eye blinks and movement artifacts removed using visual inspection and amplitude >100–200 μV. Trials with artifacts at any one of the central or mastoid electrodes.  Filters: Band passed 0.01–100 Hz. A 60 Hz notch filter was applied offline.  Reference: Referenced to the average of two mastoids  Segment length: 600 ms (-100 to 500 ms), baseline corrected  Electrodes: groups of four electrodes arranged in a 5 × 5 grid across the scalp Windows: mean amplitudes in 25–55, 85–135, 150–250, and 350–500 ms, and an additional measure 50–120 ms, after onset of stimulus, along with mean amplitude measures every 10 ms between 0 and 220 ms after onset in 30 ms epochs (i.e., 0–30, 10–40, 20–50 ms, etc.). | |
| Strait et al. (2014) | Matlab 7.5.0 | Artifacts: Muscular artifact removed using amplitudes >100 μV. Eyeblinks removed using EOG, analysis conducted using the spatial filtering algorithm in Neuroscan Edit 4.3 (Compumedics).  Filters: Band passed 0.1-100 Hz. Band passed 0.1-40 Hz.  Reference: Referenced to the average of the two mastoids  Epochs: Noise reduction algorithm applied using Pearson's correlations and the most poorly correlated 30% discarded Mean amplitudes calculated over peak maxima according to average response characteristics at Cz  Windows: First large positivity (90–130 ms), later negativity (250– 325 ms) Response variability: 300 ms post-stimulus onset, amplitude variances across 50 adjacent 6-ms increments in sub-averages computed, variances summed. | |
| Karns et al. (2015) | EEGLAB and ERPLAB | Artifacts: Movement artifacts removed using visual inspection. Eye blinks/movements removed using ERP lab and visual inspection.  Filters: Band passed 0.1-40 Hz  Reference: Relative to CMS, referenced to the average of mastoids offline.  Segment lengths: −100 to 500 ms Participants: answered <7/ 12 questions correctly in the basic comprehension questions excluded. | |
| Isbell et al. (2016) | EEGLAB and ERPLAB | Artifacts: Eye and movement artifacts removed using peak to peak >100 μV and >200 μV, followed by visual inspection.  Filters: Band passed 0.1-40 Hz  Reference: Relative to CMS, referenced to average of mastoids offline, Left and right horizontal eye channels re-referenced to one another  Segment length: -100 to 500 ms, baseline corrected  Windows: Mean amplitudes between 100 and 200 ms post-stimulus onset  Electrodes: Eight electrodes within the anterior, central, and posterior rows | |
| Wray et al. (2017) | EEGLAB and ERPLAB | Artifacts: Eye movements removed using >± 100 μV in eye channels. Movement artifacts removed using >± 200 μV and visual inspection  Filters: Band passed 0.1-40 Hz  Reference: CMS online, referenced offline to the mean of the mastoids  Segment length: −100 to 500 ms, baseline corrected Windows: Based on visual inspection of the data, mean amplitudes between 100 and 200 ms post-stimulus onset. | |
| Giuliano et al. (2018) | EEGLAB and ERPLAB | Artifacts: Movement artifacts removed using visual inspection. Eye blinks/movements removed using ERP lab and visual inspection.  Filters: high-pass filtered at 0.1 Hz. Low-pass filtered at 40 Hz.  Reference: recorded relative to the common mode sense active electrode and then re-referenced offline to the algebraic mean of the left and right mastoids  Segment: −100 to 500 msec relative to probe onset  Mean amplitudes extracted from ERPs in a 50 ms window centered on the prominent positive deflection elicited by probes in both conditions, 150- 200 ms post probe onset | |
| **Learning and memory** | | | |
| Marshall et al. (2002) | Not specified | Artifacts: Eye blinks and movements removed using ocular artifact correction and automated artifact rejection programs to regress eye blinks out of the EEG channels and movement  Filters: Band passed 0.1-100Hz Low-pass filtered at 10 Hz.  Reference: Cz during recording, ground electrode was AFz, offline re-referenced to average  Segment length: -100 to 1500 ms, baseline corrected  Windows: Mean amplitudes for 450- to 700 ms and 700- to 1350 ms for adults, and 300- to 600 ms, 600- to 900 ms, and 900- to 1,500 ms for 4-year-olds  Electrodes: ERPs from parietal and occipital sites were inverted to account for the anterior–posterior inversion effect of the average reference.  Participants: > 20 artifact-free trials | |
| Riggins et al. (2009) | Not specified | Artifacts: Eye blinks using EOG. Amplitude >±150 μV in any 100 ms window.  Filters: Not specified  Reference: Cz during recording, offline re-referenced to average mastoids  Segment length: Not specified  Windows: middle latency component and late positive potential 400–600 ms and at 900ms, broadly distributed over a 500–600 ms window | |
| Riggins & Rollins (2015) | BESA and MEGIS (GmbH) | Artifacts: Ocular artifacts correcting using an algorithm, movement artifacts removed using visual inspection, Data interpolated for <10% bad channels  Filters: Band passed 0.1-80 Hz. A 30 Hz low pass filtered.  Reference: Offline re-referenced to average  Segment length: -100 to 1500ms, baseline corrected  Windows: Nc 350-500ms and PSW 800-1000ms  Participants: <10 trials per condition excluded | |
| Canada et al. (2019) | BESA and MEGIS (GmbH) and  EEGlab (MATLAB) | Artifacts: Ocular artifacts were corrected using BESA. Segments were rejected if any artifact was detected (e.g., blink) or if the amplitude exceeded 200 microvolts (μV) or was below −200 μV.  Filters: Low-pass filtered at 0.1 Hz and high-pass filtered at 30 Hz  Reference: Re-referenced to the average of all channels  Segment length: 1600ms (-100 - 1500 ms)  Windows: Nc 350-800ms and PSW 900-1500ms  Electrodes: bilateral frontal (F3 and F4), central (C3 and C4), and parietal (P3 and P4) electrodes, and three midline (Fz, Cz, and Pz)  Bad channels were interpolated and a maximum of 10 bad channels (<15.6% channels) were allowed for inclusion in the dataset. Participants with fewer than 10 trials in any condition were excluded | |
| Meyer et al. (2014) | Not specified | Artifacts: Visual inspection  Filters: Band passed 0.016-125 Hz. A 30-Hz low-pass filter and filter padding applied.  Reference: Left mastoid during recording, offline re-referenced to average mastoids  Segment length: -200 to 1600ms, baseline corrected Windows: FRN mean amplitude 100 ms window 372 msec after feedback onset (defined based on the difference wave i.e. response to incorrect minus correct first turns)  Electrodes: frontal midline electrodes Fz, FCz, and Cz  Trials: First or second turns in which child did not attend to the screen during feedback presentation and second turns that were preceded by distraction  Participants: >10 behaviorally valid artifact free trials for each outcome category | |
| **Face processing** | | |  |
| Taylor et al. (2001) | Not specified | Artifacts: EOG or movement artifact: >150 μV  Filters: Band passed 0.1-100 Hz  Reference: Cz during recording, offline re-referenced to average  Segment length: Not specified, baseline corrected  Window: N170 determined from the grand averages of each age group (extending up to 320 ms for youngest children)  Electrodes: N170 and P1 at the posterior ± temporal electrodes P7, P8, P9 and P10 | |
| Peykarjou et al. (2013) | NetStation | Artifacts: Eye and movement artifacts removed using EOG, >150 μV followed by visual inspection  Filters: Band passed 0.1-100 Hz. A lowpass filter of 30 Hz.  Reference: Cz during recording.  Segment length: 100ms pre-stimulus, 500ms stimulus presentation, and 200ms post-stimulus, baseline corrected Windows: Peak amplitude and latency of P1, 80–180ms; N170, 130–300ms, mean amplitude of P400, 300–600ms  Electrodes: Left (66, 67, 71 and 75), midline (72, 73, 76, 77), and right (78, 83, 84, 85)  Trials: >12 bad channels excluded. Of the remaining trials, individual bad channels were replaced using spherical spline interpolation. | |
| Lochy et al. (2019) | Letswave 5 and Matlab 2012 | Artifacts: Channels with artifacts replaced using linear interpolation.  Filters: Band passed 0.1-100Hz  Reference: CMS online, offline re-referenced to average  Segment length: 2 seconds before and after each sequence, resulting in 44-second segments (-2 – 42 s). Segmented again from stimulation onset until 39.996 seconds, corresponding exactly to 48 complete 1.2Hz cycles within stimulation.  Transformation: FFT  Participants >2 rejected electrodes were excluded. | |
| Lochy et al. (2020) | Letswave 5 and Matlab 2012 | Artifacts: Channels with artifacts replaced using linear interpolation.  Filters: Band passed 0.1-100Hz  Reference: CMS online, offline re-referenced to average  Segment length: 2 seconds before and after each sequence, resulting in 44-second segments (-2 – 42 s). Segmented again from stimulation onset until 39.996 seconds, corresponding exactly to 48 complete 1.2Hz cycles within stimulation.  Electrodes: P7, P8, PO3, PO4, O1, and O2  Transformation: FFT | |
| Meaux et al. (2014) | ELAN software | Artifacts: Eye and movement artifacts removed using EOGs visual inspection.  Filters: Band passed 0.1–70 Hz. Digitally filtered (0–30 Hz).  Reference: A nose electrode during recording, offline re-referenced to average  Segment length: 1100 ms (-100 to 1000 ms), baseline corrected Window: P1 and P2 (between 90–130 and 270–310 ms, respectively), N170 (between 170 and 230 ms), all within a 40- or 60-ms time window around the peak of the grand average waveform.  Electrodes: P1, P2 at O1 and O2; N170 at T5 and T6. Participants: <50 trials per emotion excluded | |
| Melinder et al. (2010) | NetStation | Artifacts: Movement artifacts removed using >80 uV (adults) or 150 uV (children). Eye movements removed using vertical, amplitude changes > 150 uV (adults) or 200 uV (children), horizontal, amplitude >100 uV (both adults and children), Followed by visual inspection. Channels containing artifact were replaced using spherical spline interpolation.  Filters: Band passed 0.3-30-Hz  Reference: Cz during recording, offline re-referenced to average  Segment length: -100 to 800 ms, baseline corrected  Windows: P1 (child = 80-168 ms, adult = 52-120 ms), N170 (child =168-300 ms, adult =108-180 ms), and P2 (child = 260-388 ms, adult = 164-252 ms)  Electrodes: occipitotemporal and parietal sites  Trials: excluded if >12 sensors rejected, fixation on the face <200 ms | |
| Carver et al. (2003) | Averager | Artifacts: Amplitude >250 mV or a running average of activity >150 mV  Filters: Band passed 0.1-100 Hz, Digitally filtered 20 Hz  Reference: Cz during recording, offline re-referenced to average  Windows: visual inspection of the individual average waveforms  Trials: excluded if child did not attend, if >25% trials rejected, data was replaced using spherical spline interpolation  Participants: >10 channels requiring interpolation excluded | |
| **Emotional stimuli processing – faces** | | |  |
| Batty and Taylor (2006) | Not specified | Artifacts: Eye movements using vertical and horizontal EOG to reject trials with activity >±120 μV  Filters: Band passed 0.1–30 Hz.  Reference: Cz during recording, offline re-referenced to average  Segment length: 1100 ms (-100 to 1000 ms), baseline corrected  Windows: Peak latencies and amplitude of P1 was measured in six 30-ms time windows between 270 and 450 ms  Electrodes: P1 at O1, O2, PO9 and PO10; N170 at P7, P8, TP9, TP10, PO9, PO10. Mean fronto-central amplitudes were also measured at Fz, F3, F4, Cz, C3, C4, FC5, FC6 | |
| Vlamings et al. (2010) | Vision Analyser (Brain Products) | Artifacts: Eye movements and blinks: amplitude >± 75 uV.  Filters: Band passed 0.01–200 Hz. Filtered 0.1–30 Hz and an additional 10-Hz low-pass filter.  Reference: Online to the left mastoid and A2 was measured as an active electrode. Offline Re-referenced to average.  Segment length: 1500ms (-200 to 1300 ms), baseline corrected Windows: chosen through visual inspection of the grand-average waveforms  Electrodes: P1 - PO7, PO8, Oz, O1 and O2 and N170 - PO7 and PO8  Trials: during which the child was not looking at the screen were discarded. | |
| Jiang et al. (2017) | NetStation | Artifacts: Eye blinks and movements removed using horizontal and vertical EOG>100 μV.  Filters: Band passed 0.1–100 Hz  Reference: Online to the left mastoid and A2 was measured as an active electrode. Offline Re-referenced to average.  Segment length: -200 to 1000ms, baseline corrected  Transformation: Morlet wavelets  Windows: 0–100 ms, 100–200 ms, 200–300 ms, 300–400 ms, and 400–500 ms  Segments: those corresponding to incorrect responses and those with >10 channels with absolute amplitude >200 μν or a transition threshold of >100 μν excluded | |
| **Emotional stimuli processing – non-faces** | | | |
| Theall-Honey and Schmidt (2006) | James Long Company | Artifacts: Movement artifact removed using visual inspection. All channels excluded if any contained artifact.  Filters: Band passed 1-100 Hz  Reference: Cz during recording  Transformation: Not specified  Segment length: Not specified  Electrodes: left and right mid-frontal (F3, F4), central (C3, C4), and parietal (P3, P4) | |
| Cheng et al. (2014) | Neuroscan 4.4 | Artifacts: Movements and eye blinks removed using Visual inspection and transients >±100 μV.  Filters: Band passed at 100 Hz. IIR filter 0.1–30 Hz.  Reference: Referenced to average mastoids. Ground electrode on forehead  Segment length: 2300 ms (-250 to 2050ms), baseline corrected  Transformation: FFT using a Hamming window | |
| Hua et al. (2014) | Not specified | Artifacts: Eye blinks using an ocular artifact reduction procedure. Epochs with amplitude >±150 mV at any site.  Filters: Band passed 0-100 Hz  Reference: Left mastoid during recording, offline re-referenced to average mastoids  Segment length: −500 to 3000 ms, baseline corrected Windows: early (300–700 ms), middle (700–1500 ms), and late (1500–3000 ms)  Electrodes: Mean LPP amplitudes averaged posterior (Pz, P3, P4, Oz, O1, O2), central (Cz, C3, C4, CPz, CP3, CP4), and anterior (Fz, F3, F4, FCz, FC3, FC4) regions | |
| Hua et al. (2015) | Not specified | Artifacts: Eye blinks using an ocular artifact reduction procedure. Epochs with amplitude >±150 mV at any site.  Filters: Band passed 0-100 Hz  Reference: Re-referenced to average mastoids  Segment length: −500 to 3000 ms, baseline corrected Windows: early (400–1,000 msec), middle (1,000–2,000 msec), and late (2,000–3,000 msec)  Electrodes: Mean LPP amplitudes averaged posterior (Pz, P3, P4, Oz, O1, O2), central (Cz, C3, C4, CPz, CP3, CP4), and anterior (Fz, F3, F4, FCz, FC3, FC4) regions | |
| Mai et al. (2011) | Not specified | Artifacts: Eye blinks and movements: Trials with >10 bad channels exceeding 200 mV (absolute) or 100 mV (sample to sample) excluded.  Filters: Lowpass filtered below 20 Hz  Reference: Cz during recording, offline re-referenced to average  Segment length: -200 to 1000 ms, baseline corrected  Windows: Baseline-to-peak amplitude and latency of P1 in the 120–200 ms window, mean amplitude of FRN and PSW were measured in the 350–450 ms and 650–900 ms windows, respectively.  Electrodes: FRN- middle fronto-central electrodes (6, 7, 107, and 129), P1 - occipital electrodes (66, 71, 72, 77, 84, 85), PSW - central parietal electrodes (31, 37, 38, 42, 43, 48, 88, 94, 99, 104, 105, 106) | |

EOG = electrooculogram, DFT = discrete fourier transform, ICA = independent component analysis, ERN = error related negativity, FFT = fast fourier transformation, LAURA = the local autoregressive average, PCA = principal component analysis, RT = reaction time, LFN = lateral frontal negativity, CMS = common mode sense, DRL= driven right leg, LPP = late positive potential, FRN = feedback related negativity, PSW = positive slow wave
